# Being Lesbian, Gay, Bisexual, Trans, Queer, or Intersex (LGBTQI) and Christian: A Scoping Review of Theories and Constructs in Psychological Research

**DOI:** 10.1080/19317611.2024.2331806

**Published:** 2024-04-11

**Authors:** Catarina Rêgo-Moreira, Tiago Rocha-Silva, Liliana Rodrigues, Conceição Nogueira

**Affiliations:** Center for Psychology, Faculty of Psychology and Education Sciences, University of Porto, Porto, Portugal

**Keywords:** LGBTQI, Christianity, scoping review, psychological research

## Abstract

In this study, we aimed to map theories and constructs used in psychological research to address LGBTQI and Christian belonging. Through the scoping review method aligned with PRISMA—Extension for Scoping Reviews, we revised 150 studies from the psychological field between 2012 and 2022. We found conflict/negative theories, a turning point from negative to increasingly positive frameworks, exclusively positive perspectives, and broader and challenging theories. There is a gradual shift from conflict/negative perspectives to include positive and broader theories. These findings are critical to base and expand new approaches regarding LGBTQI issues and Christianity in future research.

## Introduction

The intersection between human sexuality and Christianity has centuries of troubles, incompatibilities, and non-dialogue, which are higher when it deals with LGBTQI issues (Foucault, 1978; Liboro, 2015; Rodriguez & Follins, 2012). Christian doctrine considers same-sex feelings and sexual practices sinful and unnatural.^1^ Regarding gender identity and expression, the idea that gender is a sociocultural and political construct is, for Christianity, a way of distorting God’s original plan for the sexes (Toldy & Santos, 2016). Therefore, LGBTQI^2^ people who are Christian may experience difficulties in dealing with this double belonging (Barton, 2010; Bowers et al., 2010; Dahl & Galliher, 2012; Gibbs & Goldbach, 2015; Ream & Savin-Williams, 2005; Sherry et al., 2010).

The idea of incompatibility between religious and LGBTQI identities prevailed in research (Moleiro et al., 2013). In many studies, LGBTQI people were reduced to their sexuality compared to heterosexual and cisgender religious people (Rodriguez, 2010). Acknowledging the invisibility of LGBTQI religious people, LGBTQI religious movements emerged in the USA a few decades ago. This spiked the interest in investigating the intersection between LGBTQI and religion, which began in the 1970s and has been proliferating since the 1980s and 1990s, continuing until today (Wilcox, 2006). The expansion of this research field was accompanied by a shift to a new paradigm of understanding LGBTQI people as individuals who can also have a religious affiliation or identity (Rodriguez, 2010). The proliferation of the literature brought the need to systematize knowledge through literature reviews and meta-analyses (Estrázulas & Morais, 2019; Lefevor et al., 2021; Lekwauwa et al., 2022; Rodriguez & Follins, 2012; Wilkinson & Johnson, 2020, 2021; Yarhouse & Haldeman, 2021).

Estrázulas and Morais (2019) highlighted four main themes on which the research has focused: (1) strategies for integrating religiosity/spirituality and homosexuality; (2) religiosity/spirituality as a risk factor or a protective factor for LGB people; (3) internalized homonegativity; and (4) clinical practice. Wilkinson and Johnson (2020) concluded that despite the social development toward inclusivity and equality, there remain negative experiences for religious lesbian and gay (LG) people. Wilkinson and Johnson (2021) highlighted the good and bad outcomes of following a religion for LG people. Lefevor et al. (2021) found health benefits and detrimental effects associated with religion/spirituality for LGB people. None of these recent literature reviews focused on theories or constructs used to address LGBTQI and Christianity.

Rodriguez (2010) reviewed three psychological theories commonly used in the psychological literature to explain the conflict that could arise from the intersection of sexual and religious identities: (1) the cognitive dissonance theory of Festinger (1957), (2) the stigma theory of Goffman (1963/1990), and (3) the identity conflict theory of Baumeister et al. (1985). In its original definition, cognitive dissonance is “the existence of non-fitting relations among cognitions” (Festinger, 1957, p. 3). Cognitions are “any knowledge, opinion, or belief about the environment, about oneself, or about one’s behavior” (Festinger, 1957, p. 3). Studies revised by Anderton et al. (2011) were in line with Festinger’s (1957) theory, highlighting cognitive dissonance experienced by LGB individuals, LGB couples, and family members of LGB individuals. Cognitive dissonance is higher as the conflict elements are more relevant to the person (e.g., if religious beliefs are fundamental to the individual and the person finds incongruency between LGBTQI and religion, more cognitive dissonance can arise). Anderton et al. (2011) also found that some strategies utilized to reduce the cognitive dissonance between religious beliefs and LGB issues were similar to those postulated by Festinger (1957) to solve cognitive dissonance in general.

From Goffman’s (1963/1990) stigma theory, Rodriguez (2010) highlights the *blemishes of individual character*, as it deals directly with homosexuality stigmatized by Christianity. There is a common point for all types of stigma: the existence of a trait—“a stigma, an undesired differentness from what we had anticipated” (Goffman, 1963/1990, p.15). When confronted with this trait in someone (e.g., homosexuality), others (e.g., the religious community) can see this person as not quite human and practice discrimination, through which they can purposefully or unintentionally decrease the person’s life chances (Goffman, 1963/1990). Accordingly, stigma management is crucial for the stigmatized individuals, i.e., how they deal with this discrimination (Rodriguez, 2010).

Goffman (1963/1990) suggested that when the stigmatized trait is not visible, managing stigma means managing and controlling information regarding the stigmatized characteristic by *passing*, concealing, obliterating, covering, or hiding. That is, for example, an LGBTQI Christian can choose to pass as not LGBTQI, to conceal, to obliterate, to cover, or to hide the LGBTQI belonging in the religious community. Particularly for trans people, these strategies can not be accessible when others know the trans person’s birth sex. So, religious trans people’s experiences can differ from LGB (Levy & Lo, 2013; Rodriguez & Follins, 2012).

The identity conflict theory proposed by Rodriguez (2010) is based on the original construct of identity conflict or legitimation crisis from Baumeister et al. (1985). In their work regarding identity crisis, Baumeister et al. (1985) postulated legitimation crisis or identity conflict as a serious struggle to reconcile diverse and incompatible commitments and the impossibility of choosing and acting coherently with all individual values and goals. This conceptualization could be applied to the intersection between LGBTQI and Christian double belonging as it deals with “the status of having a strong personal (and presumably emotional) commitment to two distinct identity components that become incompatible (Baumeister et al., 1985, p. 412).”

The above three theories have behind them the concept of conflict. Rodriguez (2010) defined conflict as “the tension that can arise between a gay or lesbian Christians’ sexual orientation and their religious beliefs” (Rodriguez, 2010, p. 9), due to the anti-LGBTQI language and sentiment proliferated by some Christian confessions (Rodriguez & Ouellette, 2000). Many psychological studies based on conflict theories hypothesize that all LGBTQI Christian individuals will experience conflict when they realize they belong to two incompatible identities (e.g., Barton, 2010; Borgman, 2009). Nevertheless, there are studies in which not all participants reported conflict regarding Christianity and LGBTQI (e.g., Fernandes et al., 2023; Rodriguez & Ouellette, 2000). Furthermore, some authors argued that conflict-related theories were not sufficient to address the complex experiences of LGBTQI-Christian people (Fernandes et al., 2023; Rodriguez, 2010; Rodriguez et al., 2019). Rodriguez (2010) has already suggested that identity integration and the empowerment theory are two possible alternative approaches beyond conflict.

Identity integration means combining religious and sexual or gender identities (Bowland et al., 2013). That is, joining beliefs into a new, positive whole identity where individuals view themselves as both LGBTQI and religious/spiritual (Rodriguez et al., 2019). Studies based on identity integration suggested that some LGBTQI Christians can integrate their same-sex attraction or gender identity and religious identity into their overall identity (Lapinski & McKirnan, 2013). They can use diverse strategies to promote the integration process, such as re-interpretation of the Bible and participating in LGBTQI-affirming religious churches^3^ (Rodriguez & Ouellette, 2000). Identity integration is not linear but an interactive process in which faith and sexual or gender identity development intertwine, and diverse environmental factors can intervene (Levy & Reeves, 2011).

Regarding empowerment theory, Zimmerman (2000) recovered Rappaport’s definition of empowerment as a process—“the mechanism by which people, organizations, and communities gain mastery over their lives” (Zimmerman, 2000, p. 44). An empowerment approach goes beyond alleviating a situation’s negative aspects by searching for positive ones (Zimmerman, 2000). Empowerment research identifies capabilities and connects individual well-being with the social and political environment (Perkins & Zimmerman, 1995). For example, individual, organizational, and community empowerment could occur at Christian LGBTQI-affirming organizations/churches. By participating in LGBTQI-positive religious organizations, LGBTQI Christians empowered themselves to integrate sexual and religious identities into a new positive whole. In this case, empowerment directly relates to how LGBTQI individuals recovered their religiosity/spirituality in a context of anti-LGBTQI bias from Christian conservative religious communities and anti-Christian bias from the LGBTQI communities, reclaiming a place for LGBTQI Christians (Rodriguez, 2010).

Recently, there has been a growing interest in the literature to address the intersection of multiple identities (Tarshis & Baird, 2021). Specifically, the unique and complex results the intersected identities produce in terms of power and privilege in a given socio-cultural context, as postulated by the intersectionality approach, initially conceived by Kimberlé Crenshaw (1989, 1991) regarding the intersection of race and gender for black women. Some authors have applied intersectionality to the intersection between LGBTQI, Christianity, and other categories, such as race and gender (e.g., Heard Harvey & Ricard, 2018).

Considering the use of intersectionality, identity integration, and empowerment, recent literature is applying new theories beyond conflict approaches and the psychological field, given the growing conscience regarding the complexity of LGBTQI and Christian belonging. However, since the literature reviews of Rodriguez (2010) and Anderton et al. (2011),^4^ to our knowledge, there is no systematization of theories used to address the phenomenon. Therefore, in the present scoping review, we aim to map theories and constructs used in psychological research to explain LGBTQI and Christian belonging between 2012 and 2022. We intend to contribute to advancing the research on the topic through mapping theories and constructs, which could be the basis for future research.

## Method

We chose the scoping review method for evidence synthesis because we intend to map a broad field of literature and find out the scope/coverage of the topic by showing the studies available (Munn et al., 2018), like a panoramic photograph. The scoping review method was first systematized by Arksey and O’Malley (2005), was advanced by Levac et al. (2010), and recently by Peters et al. (2021) according to the guidelines of the Joanna Briggs Institute (JBI) methodology and the PRISMA—ScR (Page et al., 2021), which guided the present scoping review.

### Research question

We defined the research question using the “PCC” (Population, Concept, and Context) mnemonic suggested by Page et al. (2021). The “P” is the LGBTQI population, the “C” is the phenomenon of interest, i.e., the theories and constructs that psychological studies used to address the intersection between being LGBTQI and Christian in the last decade, and the latter “C” corresponds to the psychological research setting. Accordingly, we will answer the following research question: “In the last decade, which theories and constructs have been used in psychological research to address the intersection between LGBTQI and Christian belonging?”.

### Inclusion and exclusion criteria

We determined the inclusion and exclusion criteria based on our research question and a previous general search in the literature on the topic. We include: (a) studies that address our phenomena of interest (theories and constructs regarding the intersection between LGBTQI and Christianity); (b) psychological research field, (c) scientific journal articles and dissertations (empirical and theoretical), case studies, and personal experience papers; (d) language written in English, Portuguese, and Spanish^5^; (e) quantitative, qualitative, and mixed methods study designs; (f) publication date between January 2012 and December 2022.

We exclude: (a) studies not directly answering our research question/phenomenon of interest (e.g., the study does not have a theory or a construct to frame the double belonging of being LGBTQI and Christian; the study does not directly address the theme; the study has no data regarding the religion or focuses her analysis on other faiths than Christianity); (b) books, book chapters, introductions to journal or journal special issues, commentaries, text opinion papers, and all types of literature reviews; (c) other languages than English, Portuguese, and Spanish.

### Search strategy

We developed the search strategy by first identifying relevant articles on the topic through an initial general search in EBSCOhost. Then, we applied the keywords and the index terms of relevant articles to develop a full search strategy in four steps: (1) dividing the relevant keywords into three research elements (LGBTQI people related keywords, Religion and Christianity related keywords, and Psychology related keywords); (2) for each research element, making correspondence between the most used keywords with the index terms from APA Thesaurus, Academic Search Ultimate, and Psychological and Behavioral Sciences Collection; (3) selecting the final keywords for our search strategy^6^; (4) adapting the search strategy for each database. We included the filter “Psychology” in the databases where this is possible (Scopus and Web of Science), and we added the word “psychology” to the search equation to guarantee this restriction in databases where this filter did not exist (Ebscohost).^7^ The detailed full search strategy is provided in supplementary material 1. For our final research, we chose the databases EBSCOhost,^8^ Web of Science, and Scopus due to the greater probability of finding psychological studies and covering a large spectrum of scientific literature. We also resorted to citation searching based on references from found articles and referrals suggested by other authors in the same field of study.

### Evidence selection

All identified records were retrieved and uploaded into *Covidence*^9^ software to start evidence selection, and duplicates were removed. Titles and abstracts were screened for assessment against the inclusion and exclusion criteria. We retrieved the documents examined as relevant in full text to *Covidence* software. Where needed, we contacted the studies’ authors by e-mail or message in research platforms (e.g., ResearchGate) to ask for the full text. Disagreements were solved through discussion between the authors of this scoping review.

### Data extraction

We created a table to facilitate the data extraction phase (Supplementary material 2). In addition to content data (theory or construct behind LGBTQI and Christian belonging), we collected details characterizing the studies analyzed, such as relevant sample characteristics (sexual or gender identity, age, and Christian affiliation), geographic context, study design, and methods. We revised and modified this tool for data extraction as many times as necessary.

### Data analysis and presentation

**Figure 1.**
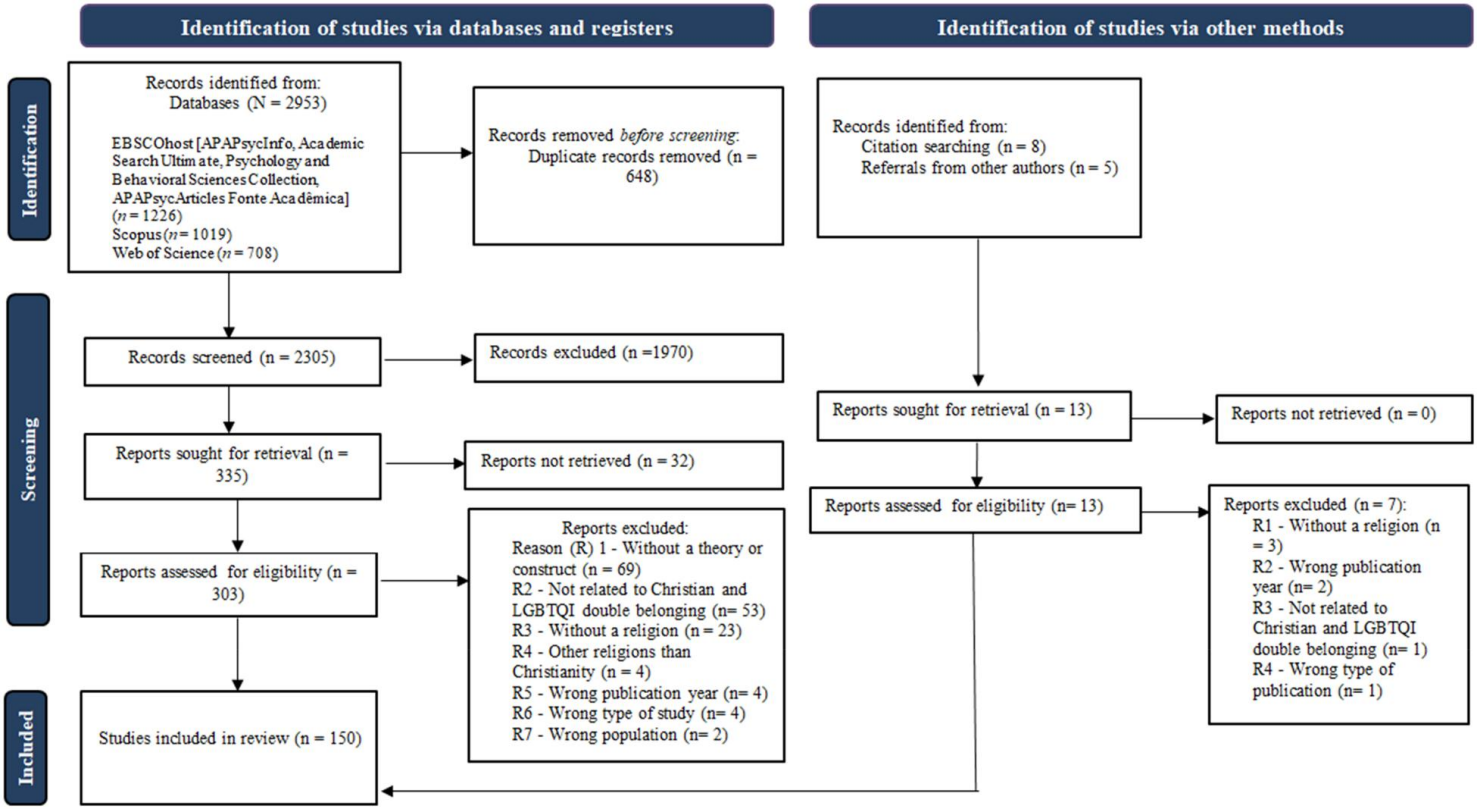
PRISMA—ScR flow diagram of the search process, study selection, and inclusion process.

**Figure 2.**
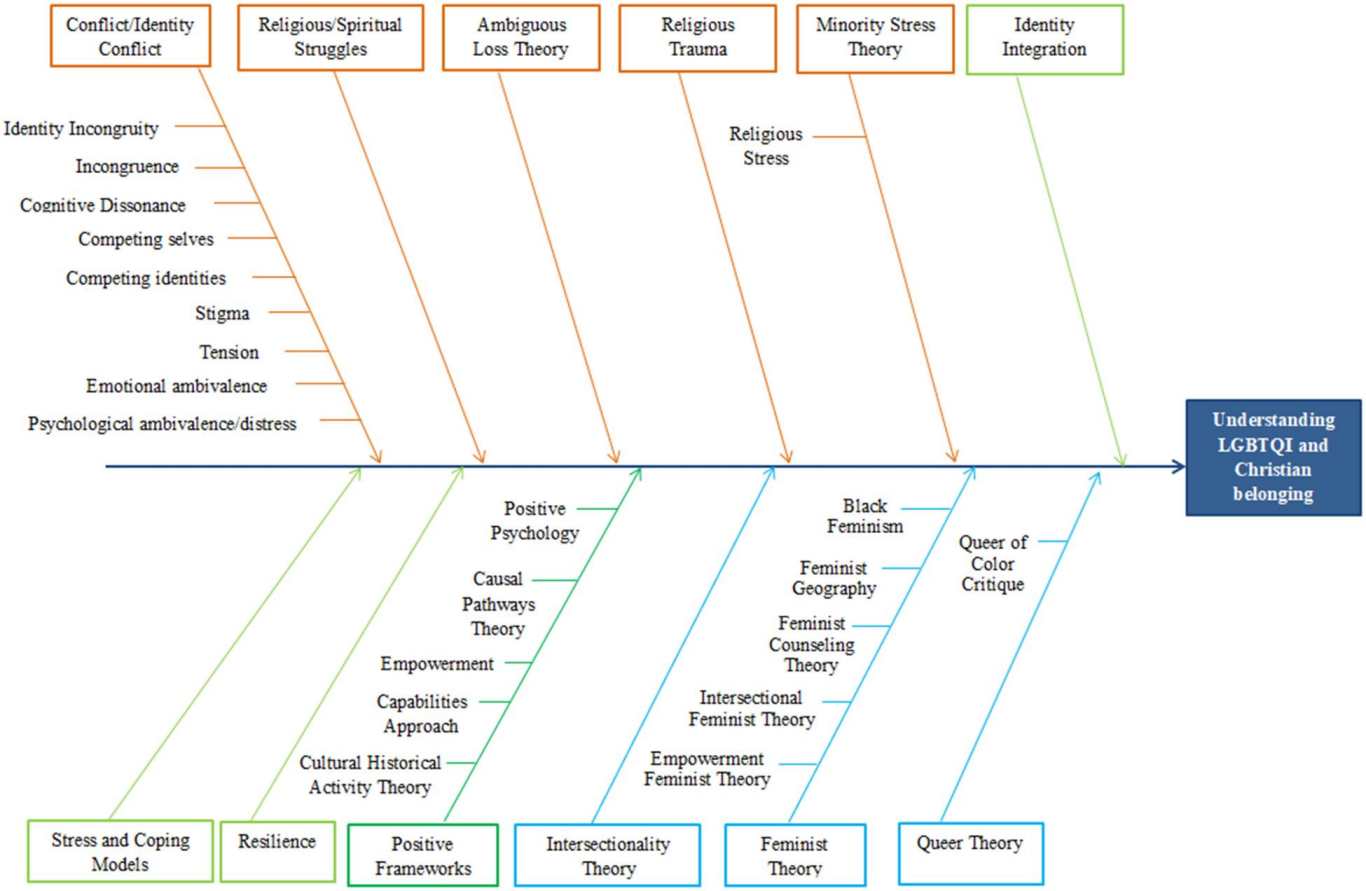
Graphical overview of core findings.

Finally, we present the data collection process through the PRISMA-ScR diagram in Figure 1. Then, we present a summary of the characteristics of the included studies. The content findings regarding our research question are in Table 1 and Figure 2, accompanied by a narrative summary according to the objectives and research question.

## Results

According to our search strategy, we identified 2953 studies through databases and 13 studies through other methods, such as citation searching and references from other authors in the field, adding up to 2966 identified studies. After duplicates were eliminated (*n* = 648), titles and abstracts were screened (*n* = 2318), and the full texts were analyzed (*n* = 316), we included 150 studies (Figure 1).

### Summary of the included studies

An overview regarding the characteristics of the included studies shows: (1) predominance of qualitative research design (*n* = 84 out 150 studies), followed by quantitative design (*n* = 53 out 150 studies), few studies using mixed methods (*n* = 6 studies), and seven theoretical studies; (2) diverse sexual and gender denominations within and beyond the LGBTQI acronym (e.g., “LG” and “LGB” corresponding to sexual identities, “men who have sex with men”—MSM—regarding sexual practices or behaviors, “same sex attracted”—SSA—referring to attraction, particularly in studies within the Mormon community, and “trans,” “transgender,” “gender-nonconforming,” and “transgender and gender-nonconforming”—TGNC—referring to gender identity); (3) the Roman Catholic Church as the most reported Christian affiliation (*n* = 78 out of 150 studies), followed by unspecified Christian affiliations (*n* = 58 studies), and the Church of Latter Day Saints (LDS)/Mormon (*n* = 29 studies); (4) most studies conducted in the USA (*n* = 122 out of 150 studies); and 5) all the included studies written in English except for one in Spanish. In supplementary material 2, we provide details regarding these characteristics.

### Content findings

In the last decade, we have noticed a gradual shift from negative to increasingly positive perspectives to address the experiences of being LGBTQI and Christian (Figure 2). This development comprises four key findings that answer our research question: (1) conflict and negative perspectives; (2) turning point from negative to increasingly positive frameworks; (3) positive approaches; and (4) broader and challenging theories. In some studies, different theories coexist. All main theories and constructs and the corresponding studies are presented in Table 1. Secondly, we found other theories used to complement the primary theoretical background. We present them as complementary theories.

#### Conflict and negative perspectives

We found a significant number of studies (*n* = 123 out of 150 studies) based on conflict and negative perspectives to address LGBTQI and Christian double belonging, which include identity conflict theory, religious and spiritual (R/S) struggles and battles, ambiguous loss theory, religious trauma, and minority stress theory. Overall, this group of theories assumes a conflict premise related to being LGBTQI and Christian and postulates mainly negative results for LGBTQI Christian individuals.

##### Conflict and identity conflict

The construct of conflict and the identity conflict theory, as defined by Rodriguez (2010) and Rodriguez and Ouellette (2000), dominates as the primary background in the studies analyzed (*n* = 81 out of 150 studies). We also found the use of terms close to conflict and identity conflict, such as tension (Best & Weerakoon, 2021; Quinn et al., 2016; Reygan & Moane, 2014), emotional ambivalence (Kay et al., 2021), psychological ambivalence (Wolff et al., 2017), competing identities (Hedge, 2017; Nadal & Corpus, 2013), competing selves (Longo et al., 2013, based on Sherry et al., 2010), incongruence (Wolff et al., 2016), and identity incongruity (R. M. Liboro, 2015; R. Liboro & Walsh, 2016). Cognitive dissonance rooted in Festinger’s (1957) work and aligned with conflict background was also found (*n* = 15 out of 81 studies).

We noticed a tendency to address not just the internal or individual conflict but also external conflict that often occurs with the Christian community to which the LGBTQI individual belongs or with people of the same Christian confession (Etengoff, 2017; Yarhouse & Carrs, 2012). Noteworthy is that some studies postulate that even if the conflict was present in their findings, it does not happen for all LGBTQI participants (Coley, 2019; Etengoff, 2014; Etengoff & Rodriguez, 2017; Yarhouse & Carrs, 2012).

##### R/S struggles and battles

Not far from the identity conflict, we found studies using the construct of R/S struggles or battles (*n* = 11 out of 150 studies). Exline et al. (2021) expose six R/S struggle types that Christian LGBTQI individuals could experience (divine, demonic, interpersonal, moral, ultimate meaning, and doubt struggle). Zarzycka et al. (2017) postulate religious struggle as a religious dimension, which includes concern with individual guilt, feeling not forgiven by God, negative emotions regarding God, and negative social interactions related to religion. Similarly to the meaning of struggle, M. Wood (2016) refers to the phenomenon of dealing with the intersection between homosexuality and religion as a “battle.” Some studies argue that R/S struggles could result in the loss of religious identity to maintain sexual identity with feelings of grief (e.g., W. Wood & Conley, 2014).

##### Ambiguous loss theory and religious trauma

Grief, loss, and bereavement appeared specifically framed by the Ambiguous Loss Theory (ALT) from Boss (2016) and by the Religious Trauma Syndrome (RTS) from Winell (1993/2007). Okrey Anderson and McGuire (2021) expanded ALT to the experiences of trans Christian people. These authors found a fundamental feature of ambiguous loss—feeling uncertain about a family’s boundaries and whether the person is inside or outside. This feeling could be experienced by trans Christian people when they lose physical access to their faith communities or when they suffer ruptures in their relationship with God or other members of their faith communities (Okrey Anderson & McGuire, 2021).

As a result of the ruptures that could occur with the religious community and the anti-LGBTQI religious messages, LGBTQI-Christian people could experience a condition close to Post Traumatic Stress Disorder called RTS (Winell, 1993/2007). RTS includes symptoms of distress that are unique to religious and spiritual conflicts and could result in prevalent psychological damage (Stone, 2013). Two studies (Crocker, 2022; Hollier et al., 2022) based on religious trauma and highlighted negative mental and physical health consequences in LGBQ participants. Hollier et al. (2022) added that minority stress and microaggression are two mechanisms behind religious trauma.

##### Minority Stress Theory

Meyer’s (2003) Minority Stress Theory postulates that the poorer results in the mental health of LGBTQI people, compared to their heterosexual and cisgender counterparts, are due to the additional stressors that LGBTQI individuals have to deal with. These stressors could be proximal (internal processes) and distal (external conditions or events). In our analysis, 29 studies were based on the Minority Stress Theory, suggesting that LGBTQI individuals in conservative religious confessions experience unique proximal and distal stressors. For example, messages about sinfulness regarding sexual and gender diversity (Puckett et al., 2018), early exposure to non-affirming LGBT Churches (Paulez et al., 2023), being raised in religious families and staying in the same religion in adulthood (Heiden-Rootes et al., 2021), and institutionalized discrimination in religious institutions (Baiocco et al., 2014) could contribute to minority stress, as distal stressors. Otherwise, acceptance concerns, concealment of identity, questioning people’s intentions, and fear of judgment could be proximal stressors related to being LGBTQI and Christian (Skidmore et al., 2022). Globally, a non-affirming religious affiliation can be a minority stress factor (Barnes & Meyer, 2012).

All of the stress factors mentioned above could be framed by the construct of “religious stress,” referred to in some of the included studies (Page et al., 2013; Schindler, 2021; Subhi & Geelan, 2012) as a specific form of stress because of the conflict that a sexual minority might feel from religion (Page et al., 2013). Noteworthy, some studies on minority stress background also found positive factors that contribute to dealing with minority stress, such as community support and a good individual relationship with God (e.g., Puckett et al., 2018). Gattis et al. (2014) highlight a risk and protective factor framework regarding the role of religious affiliation for sexual minority youth. Lomash et al. (2019), recognizing protector factors from religion to LGBTQI people but also adverse mental health outcomes related to minority stress, framed their study in an LGBTQ microaggressions framework.

#### Turning point from negative to increasingly positive frameworks

A second group of frameworks comprises identity integration, stress and coping models, and resilience (*n* = 33 studies). Mainly, studies based on these theories and constructs start from a negative situation and turn to positive possibilities (e.g., from identity conflict to identity integration or from a stressful situation to coping with that).

##### Identity integration

Some studies (*n* = 23 out of 150 studies) focused on identity integration of religion and sexuality or gender identity by LGBTQI individuals, with the concept of integration presented in the introduction or with a close one, such as “identity reconciliation” and “identity consolidation.” Reconciliation is “the process of bridging or connecting the sexual and religious identities, or perhaps balancing the two identities so that a client’s sexual and religious identities coexist more in harmony with each other (to the greatest extent possible)” (Mundell, 2017, p. 21). Radojcic (2016), studying an LGBT religious group, explains identity consolidation as the process through which groups embrace an identity that combines two apparent incompatible preexisting identities.

Identity integration is viewed as a diverse individual process (Houghton & Tasker, 2021) influenced by many factors and features, such as interpersonal factors (e.g., social support), intrapersonal (e.g., academic success or failure, sense of belonging or isolation, self-acceptance or self-rejection of own sexuality) (Snow, 2018), religious impediments (e.g., Bible passages), non-religious impediments (e.g., societal discrimination), and sociopolitical factors (e.g., social and governmental perception of homosexuality) (Tay et al., 2018). Conflict resolution could be a significant part of the identity integration process, including positive states (Tay et al., 2018), and participating in LGBT-affirming churches also positively contribute to identity integration (Grossman, 2021). Some studies highlight the positive consequences of the identity integration process on well-being (Scroggs et al., 2018) and found identity integration as a protective factor regarding guilt and shame (Anderson & Koc, 2020) and against internalized sexual prejudice (Anderson et al., 2021).

##### *Stress and coping model (from* *Park and Folkman, 1997) and other coping frameworks*

Three studies adapted Park and Folkman’s (1997) Model of Meaning in the Context of Stress and Coping to explain how LGBT individuals make meaning of their sexuality and Christianity. Bowland et al. (2013) addressed the two main concepts of the model—global meaning and situation meaning—to give insight into how LGBT individuals might integrate sexuality and religion. The situation to meaning-making is coming-out as LGBT, and the global meaning system is religion or spirituality. According to Bowland et al. (2013), the search for meaning starts as a coping process when coming out is a troublesome life event due to the disruption of the individual’s global meaning system (religion). Meaning-making operates as a form of coping to achieve transcendent or positive meaning.

Foster et al. (2015) incorporated the expansion of the model by Bowland et al. (2013) and refined it. These authors also agree that coping begins when the person accepts their sexual orientation while attempting to maintain religion (the global meaning). In the meaning-making process, existential questions emerge (e.g., Who am I? Did God create me this way?), and answering these questions is fundamental to developing spiritual resilience. At this point, Foster et al. (2015) expanded prior work—they suggest that meaning-making is a form of resilience as LGBT people redefine life’s customs and religious beliefs.

Hill (2015) referred to Pargament’s (1997) Theory on the Psychology of Religion and Coping, postulating that religion could provide positive coping strategies for stressful situations through actions of conservation and transformation. Regarding this theory, participants can protect or maintain what is significant in religion and transform values that are no longer helpful. Tillman (2022) also addressed coping mechanisms for cognitive dissonance, such as compartmentalization, moving from organized religion to spirituality, reinvestigating religion (e.g., Bibble passages), converting to an LGBT-affirming religion, and leaving religion.

##### Resilience

Six studies specifically used resilience to explain the experiences of their LGBTQI-Christian participants. Crocker (2022) advanced the construct of “spiritual resilience” as the perseverance of LGBTQI-Christians to maintain their faith identities despite the adversity they have encountered in religious contexts. According to Crocker (2022) and Foster et al. (2015), resilience is increased through three pathways: (1) reinterpreting the meaning of Bible passages frequently used against LGBTQI people, thereby transforming theology; (2) finding a safe and welcomed religious community; and (3) working for social justice by volunteering in non-governmental organizations (Crocker, 2022) and by transforming the Church from within in an empowerment perspective through occupying advocacy and leadership positions (Foster et al., 2015).

Chiongbian et al. (2023) proposed a model of faith-based resilience for LGBT, which includes: (1) developing awareness of sexuality; (2) experiencing difficulties, such as concealing sexual identity, discrimination, and social and self-rejection; (3) reconnecting to faith by joining a supportive religious community and developing deeper faith connection; (4) growing outward through the continuing deepening of faith and extending to others. Thamrin et al. (2022) framed the experiences of their LGBTQ participants in the model of risk and resilience from Luthar et al. (2000), simultaneously addressing risk factors and resilience related to religious importance and attendance.

#### Positive approaches

Our third content finding highlights studies just focused on positive approaches, such as positive psychology, causal pathways theory, empowerment, capabilities approach, and cultural-historical activity theory (*n* = 6 studies). These approaches assume a positive premise and focus their analysis on positive outcomes regarding LGBTQI and Christian belonging.

##### Positive psychology

Not all studies negatively address the intersection between LGBTQI and Christianity. Some studies identify positive aspects mixed with negative ones (Bowland et al., 2013; Lockett et al., 2023; Tay et al., 2018; Yarhouse & Carrs, 2012), such as a sense of acceptance from God, positive support, and participation in LGBT affirming churches. Other studies have exclusively explored positive outcomes (Rosenkrantz et al., 2016; Schollars et al., 2021; Wong et al., 2022). Tay et al. (2018) suggested integrating the positive aspects into the broader field of positive psychology frameworks. Schollars et al. (2021) framed their study into positive psychology, analyzing the new construct of “grace,” defined as the gift of unconditional and voluntary acceptance by an unobligated giver to an undeserving person. Despite some tensions and struggles, participants reported experiencing grace from God and the transforming nature of grace. They also reported how they maintain their relationship with God and God’s grace through nature, community, and creative expression (Schollars et al., 2021).

##### Causal pathways theory

Koenig’s (2012) Causal Pathways Theory is referred to by Skidmore et al. (2022) to explain the benefits of how LGBT Mormon participants navigate their intersecting religious and sexual identities. This theory explains how religion could promote health and well-being at three levels. First, at a psychological level, religion could increase positive coping skills and a stronger sense of purpose and life meaning. Second, religion could promote health at a behavioral level by discouraging risky behaviors and promoting prosocial actions. Lastly, at a social level, religion could provide a stronger sense of social support and belongingness.

##### Empowerment, capabilities approach, and cultural historical activity theory

We also found references to empowerment theory (Meades, 2022) and backgrounds close to empowerment, such as the capabilities approach and cultural-historical activity theory (Etengoff, 2017). As empowerment focuses on identifying capabilities and connecting individual well-being with the social and political environment (Perkins & Zimmerman, 1995), we included the capabilities approach and cultural-historical activity theory in this subsection. Etengoff (2017) refers to capabilities as the opportunity for individuals to use their abilities to endorse choices and changes within their political, social, and economic structures. Specifically, this author postulates that researchers can develop empowerment settings in discriminatory contexts by promoting participants’ internal abilities (e.g., writing petitioning letters to religious leaders). Based on Bernstein’s (1997) postulation regarding empowerment through identity, Radojcic (2016) suggested that identity consolidation in a religious LGBT group occurs through Catholic LGBT identity empowerment within the Catholic Church through actions, community building, and destigmatization.

The cultural-historical activity theory postulates that the person and their environment are an interactive unit of analysis as individuals involve themselves in “mediated actions” by appropriating and transforming artifacts, objects, and people in the search for meaning, conflict resolution, and social change. For example, LGBTQI people from religiously conservative backgrounds created Facebook and blogs that served as coming-out, community-building, and sense-making tools to manage the tensions between their sexuality, family, and “homoprohibitive” religious systems (Etengoff & Daiute, 2015).

#### Broader and challenging theories

A fourth group of studies (*n* = 30) used intersectionality, feminism, and queer theory. These frameworks have in common the trait of being broader and more challenging than the other found theories. They go beyond rigid identities and include multiple and intersected belongings. Some studies combine intersectionality with feminism, and other studies use queer theory, sometimes joined intersectionality. We also found ramifications of these theories, such as feminist geography, black feminism, feminist counseling theory, empowerment feminist theory, and queer of color critique.

##### Intersectionality, feminism, and combinations of both

Twenty-five studies addressed the intersecting LGBTQI and Christian identities based on or suggesting intersectionality theory (Crenshaw, 1989, 1991) or an intersectional framework.^10^ These studies understand Christianity and LGBTQI in the same person as an intersection of two categories that could result in more complexity than the sum of the two or each separated identity. For example, a Christian lesbian could have a different experience than someone who identifies as Christian and not lesbian or as lesbian and not Christian (Jacobsen & Wright, 2014). A Christian LGBTQI person could experience unique discrimination for being LGBTQI in religious settings (e.g., through scriptural teachings or excommunication from the religion) and also discrimination for being religious in LGBTQI settings (Skidmore et al., 2022). Some studies showed how complexity increases as more stigmatized categories intersect in the same person, for example, studies with black religious LGBTQI people (Garrett-Walker & Torres, 2017; Lefevor, Smack, et al., 2020; Wong et al., 2022) and a study with an intersex, trans woman, and Christian (Lesher, 2018).

Feminist theory appeared in five studies. In a general feminist approach, the assumption that the personal is political and the expertise of individuals in their own experiences was the most used feminist premises (Hill, 2015; Rodriguez et al., 2013). Other studies applied ramifications of feminism: (1) feminist geography (Ho & Hu, 2016) to highlight the Church as a social institution associated with specific space/power/knowledge practices; (2) black feminism (McGuire et al., 2017) to address how students’ spiritual/religious identities are raced and gendered and interact with their sexual identities; (3) feminist (counseling) theory (Parker et al., 2019) to analyze the narratives of oppression and reconciliation of lesbian women from the Pentecostal Church; and 4) empowerment feminist theory (Grimes, 2020) to explore the lived experiences of trans women raised in Christian denominations. Coburn et al. (2019) combined intersectionality with feminism in the intersectional feminist theory to address how gender intersects with other social categories, such as race and sexual orientation.

##### Queer theory

Queer theory was used in five studies. This theory allows for critical analysis, deconstruction, and resistance regarding binaries of gender and sexual orientation, defying the supposed “naturalness” of hetero-cis-normativity (Gabriele-Black & Goldberg, 2021). For example, the experiences of queer Christian women in Christian churches (Coburn et al., 2019), the process of identity exploration of queer emerging adults raised in Evangelical Christian environments (Black, 2018), and the experiences of queer youth in Evangelical Christian campuses (Gabriele-Black & Goldberg, 2021).

Levy and Harr (2018) argues that queer theory challenges the notion that one cannot be bisexual or pansexual and Christian. These authors suggested that their Christian bisexual and pansexual participants *queered* their sexual and faith identities by living authentically and moving beyond labels and assumptions. McGuire et al. (2017) framed their study regarding religious black queer people in queer of color critique (QCC). This theoretical framework was born in the same way as black feminism concerning feminism, but regarding queer theory, which was only approached from a white point of view (McGuire et al., 2017). These studies also added intersectionality to complete the theoretical background due to the multiple belongings of their participants.

**Table 1.** Main theories and constructs used to explain LGBTQI and Christian belonging.

Theory or construct	Study citation
Conflict/identity conflict	(Beagan & Hattie, 2015; Best & Weerakoon, 2021; Cerbone & Danzer, 2017; Chestna, 2015; Claybaugh, 2014; Coley, 2019; Craig et al., 2017; Crockett et al., 2018; Dahl & Galliher, 2012; Dangerfield et al., 2019; Dean et al., 2021; Deguara, 2018; Dehlin et al., 2015; Etengoff, 2017; Etengoff, 2021; Etengoff, 2014; Etengoff & Rodriguez, 2017; Fallon et al., 2013; Fernandes et al., 2023; Freeman-Coppadge and Horne, 2019; Gandy et al., 2021; Gerena, 2019; Gibbs & Goldbach, 2015, Gibbs & Goldbach, 2021; Goodrich et al., 2016; Greene et al., 2017; Hamblin & Gross, 2014; Hampton, 2021; Hanlon, 2013; Hart et al., 2019; Hedge, 2017; Hibma, 2018; Hinman & Lacefield, 2020; Ho & Hu, 2016; Hollowell, 2012; Houghton & Tasker, 2021; Jacobsen, 2013; Jacobsen & Wright, 2014; Kay et al., 2021; Killian et al., 2021; Kralovec et al., 2014; Lapinski & McKirnan, 2013; Lefevor et al., 2022; Levy & Harr, 2018; Levy & Lo, 2013; R. M. Liboro, 2015; R. Liboro & Walsh, 2016; Lockett et al., 2023; Longo et al., 2013; Mascaro, 2017; McKinney & Storlie, 2021; Meladze & Brown, 2015; Moleiro et al., 2013; Mosher et al., 2019; Myler, 2013; Nadal & Corpus, 2013; Pietkiewicz & Kołodziejczyk-Skrzypek, 2016; Quinn et al., 2016; Rabasco and Andover, 2021; Radojcic, 2016; Reygan & Moane, 2014; Rosik et al., 2022; Sadusky, 2018; Scheitle & Wolf, 2017; Sowe et al., 2014, 2017; Stamatoulakis & Nearchou, 2015; Subhi & Geelan, 2012; Tillman, 2022; Tuthill, 2016; Watkins et al., 2016; Wedow et al., 2017; Wolff et al., 2016, 2017; Yarhouse et al., 2017; Yarhouse & Carrs, 2012; Zarzycka et al., 2017)
Identity incongruity	(Liboro, 2015; Liboro & Walsh, 2016)
Incongruence	(Wolff et al., 2016)
Cognitive Dissonance (Festinger, 1957)	(Chestna 2015; Crocker, 2022; Dangerfield et al., 2019; Dehlin et al., 2015; Hart et al., 2019; Gibbs & Goldbach, 2021; Lefevor, Blaber, et al., 2020; Levy & Harr, 2018; Lockett et al., 2023; Meladze & Brown, 2015; Reygan & Moane, 2014; Sadusky, 2018; Subhi & Geelan, 2012; Tillman, 2022; Watkins et al., 2016)
Competing selves	(Longo et al., 2013)
Competing identities	(Hedge, 2017; Nadal & Corpus, 2013)
Stigma (Goffman, 1963/1990)	(Quinn et al., 2016)
Tension	(Best & Weerakoon, 2021; Quinn et al., 2016; Reygan & Moane, 2014)
Emotional ambivalence	(Kay et al., 2021)
Psychological ambivalence / Psychological distress	(Dean et al., 2021; Wolff et al., 2017)
Religious/Spiritual Struggles and Battles	(Christian, 2016; Exline et al., 2021; Mosher et al., 2019; Paul, 2019; Paulez et al., 2023; Rosa and Esperandio, 2022; Szymanski & Carretta, 2020; Wood & Conley, 2014; Wood, 2016; Zarzycka et al., 2017, 2017)
Ambiguous Loss Theory	(Okrey Anderson & McGuire, 2021)
Religious Trauma	(Crocker, 2022; Hollier et al., 2022)
Minority Stress Theory (Meyer, 2003)	(Baiocco et al., 2014; Barnes & Meyer, 2012; Craig et al., 2017; Crocker, 2022; Crowell et al., 2015; Gandy et al., 2021; Gattis et al., 2014; Greene et al., 2017; Grigoriou, 2014; Heiden-Rootes et al., 2021; Hibma, 2018; Hollier et al., 2022; Klundt et al., 2021; Lefevor, Blaber, et al., 2020; Lefevor, Skidmore et al., 2022; Lefevor, Milburn, et al., 2020; Lomash et al., 2019; Mosher et al., 2019; Page et al., 2013; Paulez et al., 2023; Puckett et al., 2018; Rosa & Esperandio, 2022; Rosati et al., 2020; Rosik et al., 2022; Schindler, 2021; Skidmore et al. 2022; Subhi & Geelan, 2012; Trecartin et al., 2022)
Religious Stress	(Page et al., 2013; Schindler, 2021; Subhi & Geelan, 2012)
Identity Integration	(Anderson & Koc, 2020; Anderson et al., 2021; Bayne, 2016; Crockett et al., 2018; Gandy et al., 2021; Goodrich et al., 2016; Grossman, 2021; Hall, 2015; Hampton, 2021; Hanlon, 2013; Hollowell, 2012; Houghton & Tasker, 2021; Lapinski & McKirnan, 2013; McKinney & Storlie, 2021; Mundell, 2017; Paul, 2019; Radojcic, 2016; Rodriguez et al., 2019; Scroggs et al., 2018; Snow, 2018; Subhi & Geelan, 2012; Tay et al., 2018; Tuthill, 2016)
Stress and Coping Model (Park and Folkman, 1997) and other Coping Frameworks	(Bowland et al., 2013; Carrico et al., 2017; Foster et al., 2015; Hill, 2015; Tillman, 2022)
Resilience	(Chiongbian et al., 2023; Craig et al., 2017; Crocker, 2022; Dahl & Galliher, 2012; Foster et al., 2015; Lefevor, Smack, et al., 2020; Walker & Longmire-Avital, 2013)
Positive (Psychology) Frameworks	(Schollars et al., 2021; Tay et al., 2018; Wong et al., 2022)
Causal Pathways Theory (Koenig, 2012)	(Skidmore et al., 2022)
Empowerment (Perkins & Zimmerman, 1995)	(Meades, 2022)
Capabilities Approach and Cultural Historical Activity Theory	(Etengoff, 2017)
Intersectionality Theory (Crenshaw, 1989, 1991)	(Barajas, 2014; Craig et al., 2017; Dangerfield et al., 2019; Gabriele-Black & Goldberg, 2021; Garrett-Walker & Torres, 2017; Gibbs & Goldbach, 2021; Grimes, 2020; Harvey & Ricard, 2018; Huffman et al., 2020; Jacobsen, 2013; Lefevor et al., 2017; Lefevor, Etengoff, et al., 2022; Lefevor, Blaber, et al., 2020; (Lefevor et al., 2018); Lefevor, Smack, et al. (2020); Lefevor, Sorrell, et al., 2020; Lesher, 2018; Levy & Harr, 2018; Meades, 2022; Quinn et al., 2016; Rodriguez et al., 2013; Rosenkrantz et al., 2016; Skidmore et al., 2022; Skidmore, 2017; Wong et al., 2022)
Feminist Theory	(Hill, 2015; Rodriguez et al., 2013)
Black Feminism	(McGuire et al., 2017)
Feminist Geography	(Ho & Hu, 2016)
Feminist Counseling Theory	(Parker et al., 2019)
Intersectional Feminist Theory	(Coburn et al., 2019)
Empowerment Feminist Theory	(Grimes, 2020)
Queer Theory	(Black, 2018; Coburn et al., 2019; Gabriele-Black & Goldberg, 2021; McGuire et al., 2017; Skidmore, 2017)
Queer of Color Critique	(McGuire et al., 2017)

*Note.* Some study citations could be repeated in the table because the studies addressed more than one theory or construct.

#### Complementary theories

Some included studies (*n* = 26 out of 150) used other theories and models to complement their primary theory or construct presented above. Ten studies complemented their understanding of LGBTQI and Christian belonging through identity developmental stage models regarding LGBT and religious/faith development. These models help to frame the process of LGBTQI and Christian belonging alongside the individual life course trajectory. This process could differ according to the sexual or gender identity development stage and the religious or faith development stage where the individual is.

Concerning sexual identity developmental models, we found studies using Cass’s (1979) homosexual identity formation model (e.g., Bayne, 2016; Scroggs et al., 2018), Troiden’s (1989) model of gay, lesbian, and bisexual identity development (e.g., Lapinski & McKirnan, 2013), sexual identity development theory from Dillon et al. (2011) (Paulez et al., 2023), and Yarhouse & Tan’s (2004) sexual identity development^11^ (Sadusky, 2018).

Some studies with trans participants referred to gender identity developmental models, such as Lev’s (2004) *transgender emergence model* (e.g., Scroggs et al., 2018), Devor’s (2004) *14-stage model of transsexual identity formation* (e.g., Levy & Lo, 2013), and Brown’s et al. (2020) gender identity development theory (Paulez et al., 2023). Arnett’s (2000) emerging adulthood identity development model appeared in two studies (Black, 2018; Scroggs et al., 2018) as a general reference framework, considering participants’ age.

Regarding religious/faith identity developmental models, we found Allport’s (1950) religious identity development (Bayne, 2016), Fowler’s (1981) faith development theory (Dahl & Galliher, 2012; Hibma, 2018; Levy & Lo, 2013; Liboro & Walsh, 2016), and Streib’s (2001) Religious Styles (Paul, 2019). Kohlberg’s (1981) moral development theory was also used (Liboro & Walsh, 2016).

The attachment to God theory from Kirkpatrick and Shaver (1992) was used in one study (Rosa & Esperandio, 2022) to suggest that the high prevalence of an avoidant attachment style in the personal relationship with God could be a strategy to deal with R/S struggles. Pescosolido and Georgianna’s (1989) network theory of suicide clarified why gay males in religious contexts are at increased risk of suicide even though they are religious (Claybaugh, 2014). Minority stress theory was complemented by the multilevel stigma theory (Herek & McLemore, 2013; White Hughto et al., 2015) and social stigma process (Frost, 2011) in one study (Paulez et al., 2023) and by the psychological mediation framework (Hatzenbuehler, 2009) in another study (Page et al., 2013).

To complement intersectionality theory, we found three concepts of Bourdieu’s theory (Bourdieu & Wacquant, 1995; Bourdieu, 2006)—field, *habitus*, and capital—(Barajas, 2014), the multidimensional identity model from Reynolds and Pope (1991) (Lesher, 2018), the identity model of Morales (1989) (Navarrete, 2020), and the concept of “personal life” by Smart (2007) (Ho & Hu, 2016, p. 1728). To better address the relationship between spirituality/religion and HIV care for transgender women of color, we noticed Andersen’s (1995) behavioral model in one study (Grimes, 2020). Regarding conflict resolution, one study (Killian et al., 2021) complemented the analysis with self-categorization theory (e.g., Turner & Reynolds, 2012), and another study (Etengoff, 2014) with relational complexity theory (e.g., Daiute, 2011).

We also found broader theories and approaches to complement primary theoretical frameworks, such as Alderson’s (2012) ecological model of LGBTI identity (Hollowell, 2012) and the postmodern constructivist approach with an evangelical evaluative framework, considering participants’ religious and sociocultural context that lead them to celibacy (Hedge, 2017). In a Psychodynamic approach, Hanlon (2013) referred to Kohut’s (1971/2009) Self Psychology Theory, and Eller-Boyko and Grace (2017) used terms from Jung’s depth Psychology (e.g., archetypal feminine) to frame the first author’s experience of being lesbian and Christian.

## Discussion

The present scoping review included 150 studies in the psychological literature, published between 2012 and 2022, related to being LGBTQI and Christian. We collected theories and constructs used in these studies to explain the intersection between LGBTQI and Christianity. Overall, the findings show a path starting from conflict and negative perspectives (identity conflict, R/S struggles, ambiguous loss theory, religious trauma, and minority stress) passed through a turning point to increasingly positive frameworks (identity integration, stress and coping model, and resilience), ending in positive approaches (positive psychology, causal pathways theory, empowerment, and cultural-historical activity theory), and broader and challenging theories (intersectionality, feminism, and queer).

Conflict and negative perspectives have a strong presence when studying the intersection of LGBTQI and Christianity (*n* = 121 out of 150 studies). This is unsurprising regarding the history and core Christian beliefs against sexual and gender diversity (Moita, 2001; Toldy & Santos, 2016). Additionally, the research has considered religion and LGBTQI issues to be incompatible (Moleiro et al., 2013), so when the two belongings co-exist, that would be negative or conflict. Nevertheless, even within negative or conflict frameworks, we noticed the inclusion of positive outcomes. This turning point started gradually with studies noticing some positive and protective factors simultaneously with negative ones (e.g., Gattis et al., 2014; Puckett et al., 2018). Afterward, identity integration allows LGBTQI-Christians to join the two belongings into their whole identity through a non-linear and dynamic process (Grossman, 2021; Houghton & Tasker, 2021; Rodriguez et al., 2019; Snow, 2018). LGBTQI-Christians can also go through a meaning-making and coping process, according to Park and Folkman’s (1997) model. At the last expansion of this model, Foster et al. (2015) introduced the construct of resilience as a positive one, to which LGBTQI-Christian people arrive at the end of the meaning-making and coping process.

The turning point to include gradually positive outcomes is consistent with literature suggesting other experiences beyond the conflict and adverse effects, defying the researchers to explore new perspectives to understand the phenomenon, which for many LGBTQI people is not just in the conflict (Moleiro et al., 2013; Rodriguez, 2010; Rodriguez et al., 2019). These findings began to highlight the complexity and fluidity of the LGBTQI-Christian experiences and to deconstruct the dichotomic vision regarding this topic, in which religion is positive versus negative, affirming versus non-affirming, or rejecting versus accepting (Puckett et al., 2018). The reality is even more complex than these binary scenarios.

Following the path to positive perspectives, we found studies exclusively reporting positive outcomes of the intersection between LGBTQI and Christianity. Some of these studies suggest or are based on positive psychology (e.g., Schollars et al., 2021; Tay et al., 2018). Thus, some studies do not start with a “conflict hypothesis.” They do not assume that all LGBTQI-Christians will experience conflict and make a linear process of choosing between one of the two belongings. This finding opens the door further to the diversity associated with the experiences of being LGBTQI and Christian, as the literature has warned when discussing conflict (e.g., Fernandes et al., 2023; Rodriguez et al., 2019). According to Tay et al. (2018), conflict theories explain only one part of LGBTQI Christian experiences because there are also positive states and outcomes, such as stress-related growth, which contributes to self-concept. Rodriguez and Follins (2012) had already argued regarding positive psychology, empowerment, and stress-related growth as psychological approaches that could contribute to advancing research on the role of religion and spirituality in LGBTQI experiences.

Our results go as far as broader, complex, and challenging theories, such as intersectionality, feminism, queer, and some combinations and ramifications of them (e.g., intersectional feminist theory, black feminism, queer of color critique). Those theories challenge the assumptions of the other approaches found in this scoping review due to their different conception regarding the (de)construction of identities and the role of social, cultural, and political circumstances and institutions in the lives of LGBTQI-Christian people. Queer theory challenges and deconstructs all identities and labels (Levy & Harr, 2018), addressing the complex experiences of participants who are Christian and outside the binary sex and gender system (Black, 2018; Coburn et al., 2019; Gabriele-Black & Goldberg, 2021). Radojcic (2016) called them *“heretical queers”* to name people and groups who identify with the LGBTQI community while they assume a position presumed as contrary to the LGBTQI community’s supposed goals by being involved in institutions that openly oppose LGBTQI equality, such as Christian conservative religious institutions.

In a feminist intersectional approach, Coburn et al. (2019) highlight that religious and LGBT identities are not inherently in conflict. Instead, they can be identities that support and foster each other. Some authors from different perspectives have shared this idea when they challenge the identity conflict approach (Fernandes et al., 2023; Rodriguez, 2010; Rodriguez et al., 2019). Nevertheless, from a feminist intersectional perspective, the assumption that religious and LGBT identities are always in conflict is explained concerning heteronormativity and heterosexism in society, according to which we have to be a single, aligned identity (Coburn et al., 2019). This way, conflict is necessary to choose one of the two identities to align with heteronormativity and religion or break away by welcoming only sexual or gender identity and leaving religion. Additionally, participants in the study of Coburn et al. (2019) rejected narratives of conflict because these narratives fixed the problem within the person. Therefore, responsibility is taken away from social and religious structures, such as non-affirming religious settings. Thus, more recent theories frame the phenomenon in a broader, institutional, social, and cultural perspective rather than burden the individual, as many psychological theories do, such as cognitive dissonance, stigma, and identity conflict.

Intersectionality theory advocates that individuals are not simply a total of the sum of their identities—they intersect multiple identities that could produce unique outcomes of oppression and privilege. As LGBTQI people negotiate their sexual and religious identities, they live in the *in-between spaces* where labels and assumptions fail to embrace the richness of their experiences (Levy & Harr, 2018). This idea is in line with other theories, such as empowerment (Rodriguez, 2010), that reclaim a place for Christian LGBTQI people, which until now has been little visible or not named. Given the phenomenon’s complexity, it is here where all theories and constructs found each other—to find a place for LGBTQI-Christians. Some studies combine different theories and add complementary approaches to embrace the complexity and intricateness of LGBTQI-Christian experiences. Overall, our findings show a significant advance in this research field. This progress can potentially decrease the crossfire between being LGBTQI and Christian as we come through new understandings of the phenomenon.

## Limitations and future directions

Even if we recognize specificities in each LGBTQI group and point out some studies that applied theories just for one group (e.g., Okrey Anderson and McGuire 2021), we cannot draw specific conclusions for each group, which may be a limitation of this study. We decided to consider LGBTQI as a whole, mainly because of the lack of literature in the field. For example, only 3 out of 150 studies included intersex individuals in small numbers—Rosenkrantz et al. (2016) reported 4% of the sample as intersex, agender, genderqueer, and other identification; Fernandes et al. (2023) referred one intersex participant, and Lesher (2018) developed a case study with an intersex person. Additionally, given the interaction between sexual orientation and gender and political power, there are times and issues when researching LGBTQI together make sense, without ever forgetting the inequalities within the diverse groups (Cole, 2009). For future research, it would be helpful to address the specificities of each group in the LGBTQI acronym, namely intersex-Christian people, which is nearly absent in the studies included.

Another limitation is that we narrowed the search strategy to psychological research. We could have enriched the study by extending the search strategy to other fields that would bring more contributions and give us an even more panoramic picture. Even so, since our field of work is psychology, we were interested in limiting the study to it. Future scoping reviews could include other research fields besides psychology to enrich the findings. Additionally, we noticed that restricting the psychological research field throughout the database filters and including the word “psychology” in the search equation could not be the best way to fulfill the objective of narrowing it to psychological research. As we underline in the method section, each database has a different functional model, so some studies are considered from the psychological field in one database, not another. This problem could lead to a few included studies not strictly from the psychological field. Future studies should test different ways to restrict the psychological research field.

Despite some limitations, this scoping review gives a great panoramic photograph regarding theories and constructs available in psychological research to address LGBTQI and Christian belonging. Future research in this field could use this study to base and expand their theoretical background to understand LGBTQI and Christian experiences better.

## Supplementary Material

Supplemental Material

Supplemental Material

## Notes

1 After the Roman Empire, with Scholasticism, “nature” or “natural” takes on a theological meaning as the will of God (Moita, 2001). Therefore, what is considered “unnatural” is understood to be against God’s will, such as same-sex feelings and sexual practices.

2 Throughout the text, we will preferably use the acronym LGBTQI to encompass a greater diversity of designations. However, this abbreviation may often appear shorter, and it may be used different terms, such as men who have sex with men (MSM) and same-sex attracted (SSA), which correspond to the terminologies used in the cited studies.

3 These settings are formal Christian religious institutions that address positive messages about homosexuality and transgender, ministering specifically to the LGBTQI community (Rodriguez & Ouellette, 2000).

4 These literature reviews did not follow a structured literature review method.

5 The Portuguese and Spanish languages were included due to the research team’s language familiarity.

6 We also acknowledge and complete with what Lee et al. (2016) found regarding LGBT search terminology.

7 It is noteworthy that each database has a different functional model, so some studies are considered from the psychological field in one database, not another.

8 EBSCOhost combines many databases from various research fields. For this scoping review, we chose the following databases due to their proximity to the Psychology field: Academic Search Ultimate, Fonte Acadêmica, APA PsycInfo, APA PsycArticles, and Psychology and Behavioral Sciences Collection.

9 *Covidence* is an online software tool to support detailed literature reviews, such as scoping reviews, systematic reviews, and meta-analyses. We used *Covidence* to help screen titles and abstracts, select full texts, and complete the PRISMA diagram.

10 Some studies do not refer to the original intersectionality theory, but they apply an intersectional framework or lens advocating some central ideas of intersectionality theory (e.g., Dangerfield et al., 2019). Thus, we also included them in this group of results.

11 Yarhouse and Tan (2004) model has the feature of being developed empirically with LGB and SSA Christian participants.
