# Supplementary material for: Being Lesbian, Gay, Bisexual, Trans, Queer, or Intersex (LGBTQI) and Christian: A Scoping Review of Theories and Constructs in Psychological Research
Source: Int J Sex Health. 2024 Apr 11;36(4):439–63. doi: 10.1080/19317611.2024.2331806 (PMC11562956; doi:10.1080/19317611.2024.2331806)
Supplement: Supplemental Material [file WIJS_A_2331806_SM8404.docx]

**Title: Being Lesbian, Gay, Bisexual, Trans, Queer, or Intersex (LGBTQI) and Christian:**

**A Scoping Review of Theories and Constructs in Psychological Research**

**Supplementary Material 2: Main characteristics of the included studies**

Table 1. Data extraction table with the main characteristics from included studies

| **Study Citation** | **Sample** | | | | | **Concept**  (the phenomena of interest)^3^ | **Geographical Context** | **Study Design** | **Methods** | **Theory or construct** |
| --- | --- | --- | --- | --- | --- | --- | --- | --- | --- | --- |
|  | *Sexual Orientation (S.O.) and/or Gender Identity (G.I.)^[[1]](#footnote-1)^* | *Age* | | *Christian Affiliation^[[2]](#footnote-2)^* | |  |  |  |  |  |
| 1 - Yarhouse & Carrs (2012) | 32 Male-to-female (MtF) Transgender | | 40 to 67 years (*M* = 55.31) | 12 Nondenomina-tional Congregation;  8 Metropolitan Community Churches;  4 Presbyterian;  3 Catholic;  2 Baptist;  1 Methodist;  1 Pentecostal. | | MtF transgender Christians´ experiences | USA | Qualitative | Data collection: Online survey;  Data analyses: Interpretative Phenomenological Analysis (IPA) | Conflict |
| 2 - Bowland et al. (2013) | 17 Lesbian  10 Gay | | 25 to 80 years (*M* = 52.5 years) | United Church of Christ;  United Methodist;  Lutheran;  Presbyterian;  Church of Christ;  Metropolitan Community Church;  Ecumenical Catholic. | | Negative and positive experiences in integrating faith and sexual orientation, and suggestions for LG Christians. | USA | Qualitative | Data collection:  Interviews;  Data analyses:  Content analyses | Stress and Coping Model adapted from Park and Folkman´s (1997) Coping/meaning-making model |
| 3 - Foster et al. (2015) | S.O.:  27 Lesbian and Gay  G.I.:  10 males;  17 females. | | 25 to 80 years (*M* = 52.5 years) | 15 United Church of Christ;  3 Methodist;  3 Metropolitan Community Church;  3 Lutheran;  2 Presbyterian;  3 Ecumenical Catholic. | | Resilience among LG Christians | USA | Qualitative | Data collection:  Interviews;  Data analysis: Content Analysis | A Model for LG Christian Spiritual Resilience adapted from the Model of Global and Situational Meaning from Park and Folkman (1997), including the adaptation of Bowland et al. (2013) |
| 4 - Meladze & Brown (2015) | 133 Gay men | | 20 to 80 years (*M* = 37.08 years) | Abrahanic faiths (45.1%), which includes Christianity. | | How religious beliefs and internalized shame predicted homonegativity, which is related to conflict and cognitive dissonance (Quantitative analysis).  How Caucasian and Asian gay men integrate their religion and homosexuality (Qualitative analysis) | Western and Asian countries (without information about specific countries) | Mixed-methods | Data collection:  Online survey with an open ended question;  Data analysis:  Statistical analysis and Thematic Analysis | Cognitive Dissonance Theory and Conflict |
| 5 - Wood (2016) | 21 participants (17 gay or lesbian; 3 bisexual; 1 “other”) | | 18 to 74 years | Christians | | The positions that gay Christians adopt in navigating both a Christian and a lesbian, gay or bisexual identity, particularly in the context of religious homophobia and hostility toward homosexuality. | United-Kingdom (UK) | Qualitative | Data collection:  Interviews;  Data analysis: Thematic Analysis | Battle / Religious Struggle |
| 6 - Quinn et al. (2016) | 30 Young, Black Men who have Sex with Men (YBMSM)  9 out of were bisexuals | | 16 to 24 years (*M* = 21) | African Methodist Episcopal;  Pentecostal;  Missionary Baptist;  American Baptist;  United Methodist;  Non-denominational. | | Experiences of homonegativity among YBMSM with a particular emphasis on the influence of the Black Church. Particularly, the attempts of YBMSM to conceal their sexuality in church. | USA | Qualitative | Data collection:  Interviews;  Data analysis: Thematic Analysis | Tension and Conflict |
| 7 - Wolff et al. (2017) | 7 Transgender and Gender-NonConforming (TGNC), all assigned female at birth. | | 18 to 35 years (*M*= 26.4 years) | 2 Catholic;  1 Lutheran;  1 Methodist;  1 Evangelical;  1 Presbyterian. | | Experiences of TGNC students who attended nonaffirming Christian Colleges and Universities (CCUs) | USA | Qualitative | Data collection:  Interviews;  Data analysis: IPA | Psychological ambivalence and Conflict;  The authors framed their results in the Social-ecological Model of Transgender Stigma adapted from White Hugto et al. (2015) |
| 8 – Wood and Conley (2014) | 1 Lesbian | | 26 years | Church of Jesus Christ of Latter-day Saints (LDS) | | How negative religious experiences can result in religious/spiritual struggles and loss of religious/spiritual identity in LGBT individuals. | USA | Qualitative | Case study | Religious/Spiritual struggles |
| 9 – Lefevor et al. (2018) | 64.721 participants:  62.3% women;  36.5% men;  0.3% Transgender;  0.9% No gender identity information;  87.4% Heterosexual  4.5% Bisexual;  2.9% Gay;  2.1% Questioning;  1.6% Lesbian;  1.5% Other. | | This study does not give information about the participant's ages. | 32% Christian non-Catholic;  18% Catholic. | | The influence of sexual identity, religious identity, and their intersection on mental health vis-à-vis power and privilege. | USA;  UK;  Canada. | Quantitative | Data collection:  Online survey;  Data analysis:  Statistical analysis | Psychological distress;  Intersectionality Theory |
| 10 – Tay et al. (2018) | 9 Gay men | | *M* = 26.6 years | 5 Christians | | How Christian and Muslim homosexual men in Singapore integrate their religious and sexual identities. | Singapore | Qualitative | Data collection:  Interviews;  Data analysis: Thematic Analysis | Identity integration;  Conflict resolution, as a great part of the identity integration process;  Positive Psychology. |
| 11 – Coburn et al. (2019) | 5 Queer women | | 21 to 35 years | 2 Non-denominational;  1 Baptist;  1 Episcopal;  1 Methodist.  All participants had previously been affiliated with Evangelical Church. | | Experiences of Queer Christian Women in Southeastern United States. | USA | Qualitative | Data collection:  Phenomenological three-interview series;  Data analysis: Thematic Analysis | Intersectional Feminist Theory;  Queer Theory. |
| 12 – Parker et al (2019) | 3 Lesbian | | 35 to 65 years (*M* = 45 years) | 3 Pentecostal | | The process of 3 lesbians with Oneness Pentecostal backgrounds who reconciled their religious beliefs and sexual orientation. | USA | Qualitative | Data collection:  Interviews;  Data analysis: Narrative Analysis | Feminist (Counseling) Theory |
| 13 – Anderson and McGuirre (2021) | 63 Transgender Youth | | 15 to 30 years (*M* = 22 years) | 30% Catholic;  30% Unspecified Christian;  24% Protestant Christian;  14% Baptist. | | Expand Ambiguous Loss Theory into the religion and religious rejection background for sexual and gender minority people. | USA;  Canada;  Ireland. | Qualitative | Data collection:  Interviews;  Data analysis: Thematic Analysis | Ambiguous Loss Theory adapted from Boss (2016) |
| 14 – Kay et al. (2021) | 7 Transgender and Gender Diverse | | 18 to 50 years | 3 ex Lutheran, Catholic and Pentecostal;  1 ex Methodist;  1 Unspecified Christian;  2 ex Catholic). | | Explore the interaction between TGGD identities and religious/ spiritual identity development. | USA | Qualitative | Data collection:  Interviews;  Data analysis: IPA | Emotional ambivalence (identified in one theme) and Psychological Conflict |
| 15 – Exline et al. (2021) | 305 Transgender and Gender-Nonconforming (TGNC) | | *M* = 31.4 years | Childhood religion was mostly Christian, with unspecified Christian affiliation. | | Struggles TGNC individuals face around religion and spirituality, types of religious/spiritual beliefs, experiences with religion, and gender identity related factors. | Geographical context:  North America;  Western Europe. | Quantitative | Data collection:  Online survey;  Data analysis:  Statistical analysis | Religious and spiritual Struggles; |
| 16 – Liboro (2015) | Not Applicable (N.A.) | | N.A. | N.A. | | Present a community level intervention project to work with LGBT individuals dealing with sexual and religious identities. | N.A. | Theoretical article | N.A. | Conflict termed in this article as Identity Incongruity |
| 17 – Pietkiewicz & Kołodziejczyk-Skrzypek (2016) | 8 Gay men | | 24 to 45 years | All Catholics;  One in transition to the Evangelical Church. | | Gay Catholics managing conflict between their sexual and religious selves. | Poland | Qualitative | Data collection:  Interviews;  Data analysis: IPA | (Identity) Conflict |
| 18 – Rosenkrantz et al. (2016) | 314 participants  G.I.:  52% Women;  32% Men;  12% Transgender;  4% Intersex, Agender, Genderqueer, and Other.  S.O.:  27% Bisexual;  23% Gay;  20% Lesbian;  19% Queer;  5% Questioning;  4% Other;  2% Pansexual;  1% Asexual. | | 18 to 68 years (*M* = 32.5 years) | 51% Unspecified Christian affiliation | | The positive aspects of identifying as both religious/spiritual and LGBTQ | USA | Qualitative | Data collection:  Online survey with an open ended question;  Data analysis:  Thematic Analysis | Intersectionality Theory |
| 19 – Wedow et al. (2017) | 30 LG students at a Catholic university:  15 male;  15 female. | | This study does not give information about the participant's ages. | Before College:  26 Catholic;  3 Protestant.  After College:  15 Catholic;  1 Protestant. | | The negotiation of sexual and religious identities by gay and lesbian students on a Catholic university. | USA | Qualitative | Data collection:  Participant Observation;  Archival research;  Interviews.  Data analysis:  Grounded Theory | Identity Conflict and Identity Negotiation/integration. |
| 20 – Snow (2018) | 22 individual blogs of LGBTQ evangelical college students | | This study does not give information about the blogger's ages. | All current or former students of Evangelical Colleges. | | How LGBTQ Christian evangelical students described their spirituality and their LGBTQ identity and explored factors influencing the intersection of spirituality and LGBTQ identity during college as expressed in the blog entries. | USA | Qualitative | Data collection:  Search blogs  Data analysis:  Content Analysis | Identity Integration, with focus on interpersonal and intrapersonal factors. |
| 21 – Freeman-Coppadge & Horne (2019) | 12 participants:  9 Gay;  3 Lesbian. | | 23 to 50 years | All Christian | | Psychological and spiritual well-being of current and former LG celibates. They used celibacy as a strategy of conflict resolution. | USA | Qualitative | Data collection:  Interviews  Data analysis:  Grounded Theory | Identity Conflict;  Conflict resolution through celibacy. |
| 22 – Rodriguez et al. (2019) | 750 participants:  295 Gay;  335 Lesbian;  129 Bisexual (53 Men; 67 Women) | | 18 to 82 years (*M* = 36 years) | Religious backgrounds:  51% Protestant;  30% Catholic,  Current religion:  43 Catholic;  78 Protestant. | | The religious and spiritual lives of LGB individuals with a focus on the integration of these identities. | USA | Quantitative | Data collection:  Retrospective secondary data analysis from the Northen California Health Study;  Data analysis:  Statistical analysis | Identity Integration |
| 23 – Hinman & Lacefield (2020) | 7 Gay men | | 19 to 58 years | Current or former Seventh-day Adventists (SDA) | | The process through which 7 gay men navigated identity formation and integration for their sexual and spiritual identities, specifically when being raised SDA. | USA | Qualitative | Data collection:  Interviews  Data analysis:  Content Analysis | Identity Conflict |
| 24 – Huffman et al. (2020) | 436 Participants:  240 Bisexual;  134 Gay;  62 Lesbian. | | 18 to 68 years (*M* = 25.21 years) | 91 Christian | | Attend to the multiple identities of LGB participants, particularly their religious spiritual identities, gender identity, and sexual orientation. | USA | Quantitative | Data collection:  Online survey  Data analysis:  Statistical analysis | Intersectionality Theory |
| 25 – Etengoff (2021) | 13 Gay men | | Christians:  *M* = 25 years | 8 Christian | | How gay students face challenges at Christian and Jewish colleges | USA | Qualitative | Data collection:  Interviews  Data analysis:  Narrative Analysis | Conflict and Conflict Resolution; |
| 26 – Houghton & Tasker (2021) | 6 Non-Heterosexual Women | | 18 to 65 years | All Catholics | | Ways through non-heterosexual women integrated their faith and sexuality. | UK | Qualitative | Data collection:  Interviews  Data analysis:  Thematic and Narrative Analysis | Identity Conflict;  Identity Integration. |
| 27 – Schollars et al. (2021) | 25 participants  GI:  17 male;  6 female;  1 Trans;  1 Not specifies.  SO:  20 Gay;  4 Bisexual or Pansexual;  1 Not specifies. | | 22 to 67 years (*M* = 29 years) | 16 Protestant;  1 Catholic, | | How Christians who identify as LGB experience divine grace. | USA | Qualitative | Data collection:  Interviews  Data analysis:  Grounded Theory | Positive Psychology |
| 28 – Paulez et al. (2022) | 644 participants  G.I.:  352 Male;  194 Female;  45 Trans:  53 Other.  S.O.:  317 Gay;  112 Bisexual;  94 Lesbian;  78 Queer;  43 Other. | | 18 to 70 years or older (*M* = 35.16 years) | 314 Current  Christian  549 Childhood Christian | | Examine the religious/spiritual and identity-related predictors of religious/spiritual struggles and life satisfaction among currently and former religious/spiritual sexual minorities. | USA | Quantitative | Data collection:  Online survey  Data analysis:  Statistical analysis | Religious/spiritual struggles  Minority Stress Theory.  Complementary theories:  Social Stigma Process (Frost, 2011);  Multilevel stigma theory (Herek & McLemore, 2013; White Hughto et al., 2015);  Sexual identity development theory (Dillon et al., 2011);  Gender identity development theory (Brown et al., 2020). |
| 29 – Lefevor et al. (2022) | 397 out of 1083 participants were Sexual Minorities  S.O.:  55.9% Bisexual/Pansexual;  25.2% Gay/Lesbian;  4% Queer/Other;  0.3% Questioning;  14.6% Same-sex Attracted.  G.I.:  74.8% Man;  22.2% Woman;  0.5% Trans;  2.5% Genderqueer. | | 20 to 50 years (*M* = 35.94 years) | 397 LDS | | How minority stress and religiosity/spirituality affect mental health, and whether religiosity/spirituality and minority stress relate to mental health differently for religiously conservative sexual minorities than for others. | USA | Quantitative | Data collection:  Online survey  Data analysis:  Statistical analysis | Minority Stress Theory |
| 30 – Lockett et al. (2022) | 78 participants:  S.O.  19 Lesbian;  20 Gay;  21 Bisexual;  10 Queer;  13 Heterosexual;  6 Pansexual;  1 Asexual;  2 Questioning;  1 Demisexual;  1 Skoliosexual (attraction to non-binary and genderqueer people)  G.I.  45 Female;  24 Male;  14 Trans;  4 Non-binary/Third gender;  3 Self-describe. | | 18 to 53 years (*M* = 26.52 years) | 37 Christian | | The experiences of LGBTQ people of color with religion and spirituality. | USA | Qualitative | Data collection:  Electronic survey with open-ended questions.  Data analysis:  Thematic Analysis | Conflict;  Cognitive Dissonance. |
| 31 – Skidmore et al. (2022) | 438 participants:  Birth Sex  161 Female;  277 Male.  GI  257 Men;  137 Women;  6 Transmen;  7 Transwomen;  26 Non-binary/Queer;  5 Other.  SO  10 Mostly Straight;  4 Bisexual;  77 Mostly Gay/Lesbian;  53 Lesbian or Gay;  188 Queer;  22 Questioning/Unsure;  5 Pansexual;  11 Fluid;  3 Asexual;  10 Heterossexual with Same-sex Attractions (SSA);  35 SSA;  13 Other. | | 20 to 40 years (*M* = 31.79 years) | 8 Catholic;  20 Christian – Mainline Protestant;  242 LDS. | | Challenges and benefits reported by Mormon sexual minority individuals into the way they navigate their intersecting identities. | USA | Qualitative | Data collection:  Online survey with one open-ended question.  Data analysis:  Thematic Analysis | Minority Stress Theory;  Intersectionality Theory;  Causal Pathways Theory (to explain benefits reported by participants). |
| 32 – Tillman (2022) | 29 participants:  G.I.  13 Male;  12 Female;  4 Gender-nonconforming.  S.O.  10 Gay;  3 Lesbian;  1 Trans;  9 Bisexual;  4 Pansexual;  2 Queer. | | 18 to 27 years old | 1 Jehovah´s Witness;  7 Catholics;  4 Lutherans;  1 Seventh Day Adventist;  14 Christians not specified. | | Coping mechanisms of LGBTQ+ individuals who have either left religion or who are still active in a religious practice. | USA | Mixed-methods | Data collection:  Interviews and Questionnaires;  Data analysis:  Statistical analysis;  Content analysis. | Cognitive Dissonance;  Coping Mechanisms to reduce cognitive dissonance. |
| 33 – Wong et al. (2022) | 10 participants  G.I.  5 Female/Woman;  1 Male/Man;  2 Genderqueer;  1 Trans;  1 Multigendered/Non-binary.  S.O.  3 Pansexual;  2 Bisexual;  2 Queer;  1 Asexual;  1 Gay;  1 Self-description. | | 20 to 33 years old. | 1 Islam and  Christian;  1 LDS. | | The intersecting identities of individuals who identify as LGBTQ+, Black, Indigenous, People of Color, and spiritual/religious. | USA and Canada | Qualitative | Data collection:  Collecting daily voice clips.  Data analysis: IPA | Intersectionality Theory;  Positive experiences (suggested Positive Psychological frameworks). |
| 34 – Gelech (2015) | 16 participants  All SSA;  All Men. | | 29 to 65 years old. | 2 SDA;  2 Christian not specified;  2 Liberal Christian;  1 Anabaptist Protestant;  1 Non-denominational  Evangelical;  1 Baptist;  1 Conservative Protestant;  1 Southern Baptist. | | How SSA Christian men retrospectively construct experiences of sexual-moral crisis and healing. | USA | Qualitative | Data collection:  Interviews  Data analysis:  Narrative Analysis | Conflict;  Conflict resolution. |
| 35 – Moleiro et al. (2013) | 471 participants  G.I.:  227 female;  242 male.  S.O.:  218 Gay;  151 Lesbian;  92 Bisexual;  8 Other;  2 Missing (not answer). | | 18 to 76 years old (*M* = 30.86 years) | Raised in a religious tradition:  317 Catholic;  12 Christian Non-Catholic.  Currently:  52 Catholic;  7 Christian Non-Catholic (*n* = 7). | | The relationship between LGB identity  and religion/spirituality in Portugal. | Portugal | Quantitative | Data collection:  Online and print survey  Data analysis:  Statistical analysis | Conflict |
| 36 – Fernandes et al. (2021) | 126 participants  G.I.:  80 Female;  45 Male;  1 Intersex;  1 Transman;  2 Non-binary;  1 Genderfluid.  S.O.:  47 Lesbian;  40 Bisexual or Pansexual;  38 Gay. | | 18 to 66 years old (*M* = 29.41 years) | Religious preference:  21 Catholic Christianity;  11 Non-Catholic Christianity. | | The relation between the conflict between religious/spiritual and LGB identities and subjective  well-being, dimensions of LGB identity and outness. | Portugal | Quantitative | Data collection:  Online survey  Data analysis:  Statistical analysis | Conflict |
| 37 – Grossman (2021)  **Note:** This dissertation was finished in 2020, but the recommended year citation is 2021. | 6 participants  G.I.:  4 Women;  2 Men.  S.O.:  2 Lesbian;  2 Gay;  1 Bisexual;  1 Pansexual. | | 18 to 25 years old. | 3 Christians | | The ways in which young LGB adults view how belonging to affirmative and welcoming religious places of worship and social groups influenced their self-acceptance and ability to integrate their sexual and religious identities. | USA | Qualitative | Data collection:  Interviews  Data analysis:  Thematic Analysis | Identity Integration |
| 38 – Hampton (2021) | 7 participants  GI  4 Women;  3 Men.  S.O.:  All L, G or B. | | 30 to 60 years old. | All Christians. | | How psychologists who identify as LGB and Christian hold these identities together and whether graduate training in Psychology influenced how an individual integrates the identities of being LGB and Christian. | USA | Qualitative | Data collection:  Interviews  Data analysis:  Thematic Analysis | Conflict;  Identity integration. |
| 39 – Eller-Boyko & Grace (2017) | 1 Lesbian | | No information | Christian | | Personal life report from the first author regarding being a lesbian and Christian. The second author heard and wrote about it. | USA | Qualitative | Interview | The authors use some terms from Jung´s depth Psychology (e.g. archetypal feminine), which was helpful to the first author. It is a report about a personal journey. |
| 40 – Lefevor et al. (2020) | 1128 participants  Gender:  23.9% Women  69.6% Man  1.7% Transgender  4.8% Other  Sexual Identity:  40.1% L or G;  36.7% Heterosexual ou SSA or “ex-gay”;  13.2% Bisexual;  10% Other. | | 18 to 70+ years | All current or former LDS | | Explore the assumptions of cognitive dissonance and minority stress theories as they apply to religiousness and beliefs about sexuality among sexual minority Mormons and former Mormons. | USA | Quantitative | Data collection:  Online survey  Data analysis:  Statistical analysis | Minority Stress Theory;  Cognitive Dissonance;  Intersectionality. |
| 41 – Anderson & Koc (2020) | 183 men attracted to men:  177 G or B;  6 Other. | | *M* = 29.31 years | 149 Christian; | | Identity integration as a protective factor against guilt and shame for religious gay men. | USA | Quantitative | Data collection:  Online survey  Data analysis:  Statistical analysis | Identity integration (to solve identity conflict) |
| 42– Anderson et al. (2021) | 178 men attracted to men:  173 G or B;  2 “mostly straight”;  1 “bicurious”;  1 demisexual to pansexual;  1 Pansexual. | | 18 to 63 years;  *M* = 29.32 years | 74 Christian not specified;  44 Catholic;  4 LDS;  3 Jehovah´s witness. | | Identity integration (and multiple group membership) as a protective factor against internalized sexual prejudice for religious gay men. | Australia | Quantitative | Data collection:  Online survey  Data analysis:  Statistical analysis | Identity integration (to solve identity conflict) |
| 43 – Greene et al. (2017) | 103 participants  66 heterosexuals;  31 Gay;  6 Bisexual. | | 35 to 82 years old;  *M* = 58 years. | All Christian Catholics. | | Exploration of contributions to psychological distress from sexual identity, stress, social support, and fear of compassion in Roman Catholic Priests. | USA | Mixed-methods | Data collection:  Online survey with an open ended questions;  Data analysis:  Statistical analysis and Thematic Analysis | Minority Stress Theory and Cognitive Dissonance |
| 44 – A. Dahl & Galliher (2012) | 19 participants  S.O.:  7 Gay;  3 Lesbian;  6 Bisexual;  2 Heterosexual;  1 Pansexual.  G.I.:  8 female;  8 male;  3 Trans male. | | 15 to 24 years old. | 16 LDS;  2 Catholic;  1 Presbyterian. | | Examine the interplay between sexual minority religious and sexual identity trajectories. | USA | Qualitative | Data collection:  Interviews;  Writing Journals;  Focus Groups.  Data analysis:  Thematic and Content Analysis. | Intersectionality;  Conflict. |
| 45 - Subhi & Geelan (2012) | 20 participants  10 Gay;  10 Lesbians. | | 20 to 51 years old; *M* = 36.5 years old. | 5 Catholic;  4 Anglican;  2 Metropolitan Community Church;  1 Nondenominational Christian;  1 Presbyterian;  1 Pentecostal. | | Explore the experiences of gay men and lesbians with respect to conflicts between their Christianity and their homosexuality. | Australia | Qualitative | Data collection:  Interviews  Data analysis:  Thematic Analysis | Conflict/Identity conflict and identity integration;  Cognitive Dissonance. |
| 46 – Page et al. (2013) | 170 participants  G.I.:  45% Female;  55% Male.  S.O.:  31% Lesbian;  47% Gay;  14% Bisexual female;  8% Bisexual male. | | 14 to 24 years old;  *M* = 19.5 years) | 33% Catholic;  35% Other Christian. | | Religious stress, gay-related stress, sexual identity, and mental health outcomes in LGB adolescents and emerging adults. | USA | Quantitative | Data collection:  Online survey  Data analysis:  Statistical analysis | Religious stress (as a construct), based on identity conflict theories and Minority Stress Theory (Meyer, 2003).  Complementary theory:  Psychological mediation framework (Hatzenbuehler, 2009) |
| 47 – Rodriguez et al. (2013) | 120 participants  G.I.:  67 (55.8%) females;  53 (44.2%)  males. | | 18 to 82 years old;  *M* = 32.27 years. | 15% Christian:  11 Protestants;  4 Catholics;  3 Unspecified Christian denomination. | | Examine bisexuality as it intersects with political and religious/spiritual identities. | USA | Quantitative | Data collection:  Archival secondary analysis of data from the Northern California Health Study (NCHS).  Data analysis:  Statistical analysis | Feminist theory and intersectionality |
| 48 – Walker & Longmire-Avital (2013) | 175 participants  GI:  76 (43.4%) women;  99 (56.6%) men.  50 (66%) Lesbian;  66 (67%) Gay;  26 (34.2%) Bisexual women;  33 (33.3%) Bisexual men. | | 18 to 25 years old;  *M* = 21.34 years. | 104 (59.8%) Christian not specified. | | Examine the relations between religious faith, internalized homonegativity, and resiliency for Black LGB emerging adults. | USA | Quantitative | Data collection:  Online survey  Data analysis:  Statistical analysis | Resilience |
| 49 – Jacobsen & Wright (2014) | 23 participants  GI:  All women.  S.O.:  All report experiencing same-sex sexuality at some point of their lives.  2 Bisexual;  1 Heterosexual;  1 in process of understanding her sexuality. | | 20 to 56 years old  *M* = 37 years. | All participants were some affiliation with LDS Church currently or in the past.  Currently:  Mormon or LDS (*n* = 11);  Other or no religious affiliation (*n* = 12). | | Experiences of same sex attracted women within the Mormon religion. | USA | Qualitative | Data collection:  Interviews  Data analysis:  Phenomenological methods | Conflict  Intersectionality |
| 50 – Beagan & Hattie (2015) | 35 participants  G.I.:  11 Man;  19 Woman;  4 Trans/queer;  1 Other.  SO:  10 Gay;  11 Lesbian;  4 Bisexual;  1 Heterosexual;  7 Queer;  2 Other. | | 20 to 68 years old | 30 Raised Christian  Current beliefs:  7 Christian. | | Explore how LGBTQ individuals experienced and perceived religion and spirituality. In particular, examining potential conflicts, how people sought to resolve conflicts for a coherent sense of self, and how they experienced the place of spirituality and religion in LGBTQ communities. | USA | Qualitative | Data collection:  Interviews  Data analysis:  Thematic analysis | Conflict  Identity integration (as a suggestion to solve conflict) |
| 51 – Gibbs & Goldbach (2015) | 2949 participants  G.I.:  2230 (75.6%) Male;  644 (21.8%) Female;  21 (0.7%) Transgender Male;  27 (0.9%) Transgender Female;  27 (0.9%) Other.  S.O.:  1814 (61.5%) Gay/Lesbian;  788 (26.7%) Bisexual;  285 (9.7%) Questioning;  57 (1.9%) Other;  5 (0.2%) Heterosexual. | | 18 to 24 years old;  *M* = 20.07 years. | 549 (18.7%) Christian Protestants ;  422 (14.4%) Catholics;  28 (1%) LDS;  347 (11.8%) Other Christian. | | Investigate the relationships among religious and sexual identity conflict, internalized homophobia, and suicidality amongst LGBT young adults. | USA | Quantitative | Data collection:  Online survey  Data analysis:  Statistical analysis | Identity Conflict |
| 52 – Etengoff (2014) | 50 participants:  23 gay men;  15 religious family members;  12 clinicians.  Note: In this table, we will consider only gay men participants and exclude family members and clinicians, which are not our research interest.  GI:  All men;  SO:  All gay. | | 18 to 35 years old;  *M* = 25 years. | 7 LDS;  2 Catholic;  2 Methodist;  1 Evangelical;  1 SDA. | | The conflicts that may emerge within intrapersonal, interpersonal, metaphysical (e.g. between God/morality and man), and intersystem contexts for gay men and their religious families and the cultural tools that they use to mediate conflicts. | USA | Qualitative | Data collection:  Interviews  Data analysis:  Narrative analysis | Conflict  Vygotsky’s (1934/1978) cultural historical  activity theory;  Relational complexity theory (Daiute, 2012). |
| 53 – Hollowell (2012) | 14 participants  GI:  All men;  SO:  All gay. | | 25 to 71 years old. | 3 Anglican;  1 Christian Missionary Alliance;  4 Presbyterian;  3 Catholic;  3 United Church of Canada. | | Explore the experience of spirituality in the lives of gay men. | Canada | Qualitative | Data collection:  Interviews  Data analysis: IPA. | Identity conflict; Identity integration.  Complementary theory:  Alderson´s (2012) ecological model |
| 54 – Bayne (2016) | Not applicable (N.A.). | | | | | Explore the intersection of sexual and religious identity in the college student population, to highlight unique challenges of development and potential interventions. | N.A. | Theoretical article, with a case example. | N.A. | Identity integration  Complementary theories: Cass's (1979) sexual identity developmentmodel and Allport's (1950) religious identity development model. |
| 55 – Radojcic (2016) | Not specified  **Note:** This study occurred within an LGBT catholic group/movement (Dignity); 26 elements were interviewed, but their demographic characteristics were not described in the article, neither the number nor demographic characteristics of the rest of all Dignity´s members that were observed during this study. | | | | | Address the specific ways groups go about consolidating seemingly conflicting identities, and the ways these identities can be leveraged to create social change. | USA | Qualitative – Ethnographic case study of a group. | Data collection:  Participant observation; Interviews;  Content analysis of Dignity´s literature.  Data analysis:  It was not specified for all data collection techniques. | Based on Bernstein´s (1997) assertion that identity can be used for empowerment, as a goal, or as part of a strategy. |
| 56 – Tuthill (2016) | 15 participants  All self-identified as lesbians at the time of the study. | | 28 to 53 years old;  *M* = 38.9 years | | All Catholic | Examine how Hispanic lesbian mothers negotiate their catholic religious identity with aspects of their sexual identity. Specifically, examine the strategies that Hispanic lesbian mothers use to reconcile or navigate perceived conflict between their roles as a catholic and as a lesbian. | USA | Qualitative | Data collection:  Interviews;  Data analysis:  Grounded Theory | Conflict and integration/negotiation |
| 57 – Wolff et al. (2016) | 213 participants  G.I.:  91 (43%) Male;  109 (51%) Female;  12 (6%) Transgender/other.  S.O.:  119 (56%) Gay or Lesbian;  51 (24%) Bisexual;  11 (5%) Questioning;  7 (3%) Heterosexual;  26 (12%) Other. | | *M* = 22.5 years old. | | 133 (62%) Christian not specified;  14 (7%) LDS. | Sexual minority who identify as Christian or Mormon, or attend and Evangelical or Mormon Non-affiming religiously affiliated universities will have the most psychological distress. | USA | Quantitative | Data collection:  Online survey;  Data analysis:  Statistical analysis. | Incongruence (consistent with studies about conflict) |
| 58 – Cerbone & Danzer (2017) | 1 participant  G.I.:  Man  S.O.:  Gay | | 52 years old. | | Catholic | A case summary of the treatment of a gay man who found solace and strength in his catholic faith while enduring the condemnation that the same church leveled at him for homosexuality. | USA | Qualitative | Case study | Conflict |
| 59 – Scroggs et al. (2018) | 961 participants;  G.I.:  46% Women;  46% Men;  4% Transgender;  4% Another gender identity.  S.O.:  39% Gay;  24% Lesbian;  19% Bisexual;  18% Another sexual orientation. | | 18 to 24 years old | | 38% Catholic;  14% Protestant. | The integration of religious and gender/sexual identities will increase an individual´s sense of well-being nad suggest that distancing oneself from an important religious identity may actually not be the best way to increase well-being in emerging gender/sexual minorities religious adults. | USA | Quantitative | Data collection:  Online survey;  Data analysis:  Statistical analysis with secondary data. | Raised in developmental theories (Arnett´s Emerging Adult Identity Development in general, and Cass´s (1979) model of homosexual identity development, and Lev´s (2004) transgender emergence model, in particular; and religious identity development) and cognitive consistency theories in social psychology (e.g. Festinger, 1957). To reach identity integration of gender and sexual minority and religious identities (Rodriguez & Ouellette, 2000). |
| 60 – Crockett et al. (2018) | 25 participants  S.O.:  12 Gay;  13 Lesbian. | | 25 to 60 years old (*M* = 42.92 years) | | 18 (72%) with current Christian religious affiliation. | Understanding sexual and religious/spiritual identity development and integration through investigating the lived experiences of LG individuals who grew up in religious environments. | USA | Qualitative | Data collection:  Interviews;  Data analysis:  Phenomenological approach. | Conflict and identity integration |
| 61 – Gandy et al. (2021) | 30 participants  G.I:  36.7% (11) Cisgender woman;  33.3% (10) Cisgender man;  13.3% (4) Transgender man;  10% (3) Transgender woman;  6.7% (2) Gender queer.  S.O.:  40% (12) Gay or Lesbian;  26.7% (8) Bisexual;  13.3% (4) Queer;  10% (3) Pansexual;  6.7% (2) Heterosexual;  3.3% (1) Asexual. | | 18 to 63 years old | | 12 (40%) Christian not specified;  5 (16.7%) Non-Denominationa;  4 (13.3%) Methodist;  4 (13.3%) Baptist;  4 (13.3%) Catholic);  3 (10%) United Church of Christ;  1 (3.3%) Christian Church Reformed;  1 (3.3%) Christian Universalist. | Examining why LGBTQ+ people stay in their faith communities and what experiences they have generally in those communities. | USA | Qualitative | Data collection:  Interviews;  Data analysis:  “Sort and Sift, Think and Shift” Method (Maietta, 2006). | Minority Stress Theory (Meyer, 2003) (mentioned in the theoretical framework) |
| 62 – Hart et al. (2019) | 7 participants  G.I.:  All men.  S.O.:  All gay. | | 24 to 70 years old | | 2 Southern Baptist;  1 Christian not specified;  1 Catholic. | The process by which gay men constructed spiritual identities outside the domain of institutionalized religion. | USA | Qualitative | Data collection:  Interviews;  Data analysis:  Grounded Theory | Based on conflict and cognitive dissonance related to the religion of origin, the authors point out reconciliation through seeking a path of spirituality instead of remaining within the faith of origin or identifying with another organized religion (so, the development of spirituality as a strategy to reconcile faith and sexual identity). |
| 63 – Chiongbian et al. (2021) | 14 participants  G.I.:  8 Cisgender men;  6 Cisgender women.  S.O.:  8 Bisexual;  4 Gay;  1 Pansexual;  1 Asexual. | | 18 to 24 years | | All Catholic | Queer experiences of adversity and resilience within Catholicism. | Philippines | Qualitative | Data collection:  Interviews;  Data analysis:  Narrative analysis | Resilience |
| 64– Scheitle & Wolf, (2017) | Sexual Identity:  127 Gay, lesbian, or homosexual (1.59%);  153 Bisexual (1.80%);  6965 Heterosexual or straight (96.61%).  Sex:  3275 Male (46.30 %); 3970 Female (53.70%). | | *M* = 45.91 years old | | Religious tradition at 16:  2393 (34.91%) Catholic;  1507 (19.90%) Evangelical Protestant;  1204 (16.06%) Mainline Protestant;  651 (8.49%) Black Protestant;  Current religious tradition:  1705 (25.38%) Catholic;  1331 (17.93%) Evangelical Protestant;  988 (12.93%) Mainline Protestant;  525 (6.77%) Black Protestant. | How the religious upbringing of individuals identifying as sexual  minorities compares to heterosexuals; and whether self-identified sexual minorities are more  likely to have reaffiliated away from more traditional or conservative religious  traditions or more likely to have disaffiliated from religion entirely. | USA | Quantitative | Data collection:  Online survey (General Social Surveys – a repeated cross-sectional survey of U.S.A. adults);  Data analysis:  Statistical analysis | Identity Conflict |
| 65 – Nadal & Corpus (2013) | 24 participants  G.I.:  14  Female (1 transgender MtF);  10 Male.  S.O.:  All  of the men identified as gay;  12 women identified as lesbian;  2 Queer. | | *M* = 32 years old (females); *M* = 28 years old (males). | | All participants were raised in catholic faith. | The experiences of  (LGB) Filipino Americans | USA | Qualitative | Data collection:  Focus groups;  Data analysis:  Consensual Qualitative Research (CQR). | Competing identities (that could not be reconciled) - Conflict |
| 66 – Lapinski & McKirnan (2013) | 84 participants  G.I.:  44 Male (52.4%);  39 Female (46.4%);  1 NA (1.2%).  Sexual history:  48 Sex with both genders (57.1%);  36 Sex with same gender only (42.9%). | | 18 to 55+ years old | | 62 (73.8%) Christian not specified;  24 (28.6%) Catholic;  25 (29.8%) Mainline Protestant. | The extent to which a Christian upbringing may inhibit  same-sex attracted individuals from accepting an LGB identity. | USA | Quantitative | Data collection:  Online survey:  Data analysis:  Statistical analysis | Conflict and identity integration along the identity developmental process in LGB individuals based on Troiden´s (1989) model of gay, lesbian, and bisexual identity development. |
| 67 – A. L. Dahl & Galliher (2012) | 19 participants  SO:  7 Gay;  3 Lesbian;  6 Bisexual;  2 Heterosexual;  1 Pansexual.  GI:  8 female;  8 male;  3 Trans male. | | 15 to 24 years old. | | 16 LDS;  2 Catholic;  1 Presbyterian. | The positive and negative experiences of sexual minority adolescents and young adults coming  out within a Christian religious context. | USA | Qualitative | Data collection:  Interviews;  Writing Journals;  Focus Groups.  Data analysis:  Thematic and Content Analysis. | Conflict;  Resilience.  Complementary theory:  Fowler´s (1981) faith development theory |
| 68 – Levy & Lo (2013) | G.I.:  3 Transgender;  1 Transsexual;  1 Genderqueer. | | 18 to 53 years | | Religious upbringing:  2 Presbyterian  and Baptist;  1 Church of Christ;  1 Catholic;  1 Methodist.  Current religion:  1 Christian not specified;  1 Nondenominational Christian;  1 Church of Christ;  1 Catholic;  1 Agnostic and  Christian. | The process by which individuals with a Christian upbringing resolve conflict between  their gender identity and religious beliefs. | USA | Qualitative | Data collection:  Interviews;  Data analysis: Constructivist Grounded Theory | Conflict and conflict resolution, from which the authors developed a Five-Fluid Stage Model of Resolving Conflict.  Complementary theoretical backgrounds:  Gender Identity Development (Devor, 2004 14-stage model of transsexual identity formation);  Fowler’s (1981)  faith development theory;  Postmodern Identity Perspectives;  Queer Theory. |
| 69 - Levy & Harr (2018) | 17 participants  S.O.:  7 Bisexual;  7 Pansexual;  2 attracted to all genders;  1 Bisexual and Queer.  G.I.:  4 male;  9 female;  1 Agender;  1 mostly male;  1 non-binary;  1 Transgender. | | 19 to 64 years (*M* = 36 years) | | Faith upbringings:  Baptist;  Catholic;  Church of Christ;  Episcopal;  Methodist;  Mormon;  Nondenominational;  Pentecostal;  Presbyterian;  Salvation Army;  Southern Baptist.  Current Faith:  Christian. | The process by which bisexual and pansexual individuals with a Christian upbringing resolved conflict between sexual identity and religious beliefs. | USA | Qualitative | Data collection:  Interviews  Data analysis:  Grounded Theory | Identity Conflict and Dissonance;  Process of resolution/integration; Queer theory;  The authors suggest the importance of intersectionality. |
| 70 – Lefevor et al. (2017) | 12825 participants  G.I.:  67.1% Female;  31.9% Male;  0.6% Self-identify;  0.4% Transgender.  S.O.:  85.5% Heterosexual;  4.6% Bisexual;  2.9% Gay;  2.7% Questioning;  2.4% Self-identify;  1.9% Lesbian. | | Emerging adults | | 33.4% Christian not specified;  19.3% Catholic. | The relationship between sexual identity, religious identity, and their intersection and therapeutic outcomes. | USA | Quantitative | Data collection:  Online survey:  Data analysis:  Statistical analysis | Intersectionality |
| 71 – Meades (2022) | 6 participants  G.I.:  3 Female;  3 Male.  S.O.:  3 Lesbian;  3 Gay. | | 21 to 60+ years old | | Christianity Catholicism:  1 Catholic;  1 Evangelical;  Christianity Protestantism:  1 Evangelical;  1 Church of England. | Explore lesbian and gay people’s experiences of Christianity, Islam, and  Judaism, and the implications for psychotherapy. | UK | Qualitative | Data collection:  Interviews  Data analysis: IPA | Intersectionality;  Empowerment. |
| 72 – Rosati et al. (2020) | 266 participants  G.I.:  94% Cisgender:  123 (46%) men 126 (47%) women.  6% Transgender/nonbinary/genderqueer.  S.O.:  Women - 77% lesbian; 20% bisexual; 3% queer, pansexual,  or fluid;  Men - 97% gay; 3% bisexual;  Transgender/non-binary - 86%queer, pansexual, or fluid; 14% bisexual. | | 20 to 80 years old (*M* = 41.15 years) | | 75 (28%) Catholic | Coming-out experiences of three  generations of Italian LGBQ+ people, namely examining the coming-out process within the religious  context and its effect on participants’ experiences of minority  stress. | Italy | Quantitative | Data collection:  Online survey:  Data analysis:  Statistical analysis | Minority Stress Theory (Meyer, 2003) |
| 73 - Gibbs & Goldbach (2021) | 46 participants  Gender:  17 Female (37%);  17 Male (37%);  2 Transfemale (4%);  7 Transmale (15%);  3 Queer (7%).  S.O.:  7 Lesbians (15%);  11 Gay (24%);  12 Bisexuals (26%);  8 Pansexuals (17%);  7 Other (15%);  1 Asexual (2%). | | 14 to 19 years old  (*M* = 16.26 years) | | Religious affiliation of origin:  17 (37%) Catholic;  16 (35%) Christian not specified;  1 (1.2%) LDS;  Current religious identification:  8 (17%) Catholic;  7 (15%) Christian not specified. | Exploring negative messages that  sexual minority adolescents receive from religious sources, the identity dissonance experience that  may occur, and strategies that youths use to make sense of antihomosexual  religious messages using a cognitive dissonance framework. | USA | Qualitative | Data collection:  Interviews  Data analysis:  Thematic analysis | Cognitive Dissonance Theory  (with an intersectional approach) |
| 74 – Lomash et al. (2019) | 90 participants  G. I.:  39% Male;  37% Female;  9% Genderqueer;  7% Gender Non-Conforming;  6% Trans;  2% Third Gender;  Agender 1%.  S.O.:  31% Bisexual;  23% Gay:  16% Lesbian;  14% Queer;  12% Pansexual;  2% Heterosexual;  1% Asexual. | | 18 to 58 years old  (*M* = 27.9 years) | | 16% Christian Protestant;  15% Other; 10% Catholic;  8% Unitarian Universalist; 6% Christian – Nondenominational;  4% Christian not specified. | How LGBTQ  individuals experience microaggressions that are framed in a religious or spiritual  context | USA | Qualitative | Data collection:  Survey with open-ended questions;  Data analysis:  Thematic analysis | Microaggressions framework related to minority stress |
| 75 – Killian et al. (2021) | N.A. | | N.A. | | N.A. | Queer-religious-spiritual clients’ identity negotiation processes and intersections. | USA | Theoretical article | N.A. | Conflict;  Complementary theory:  Self-categorization theory (e.g., Turner & Reynolds, 2012) |
| 76 – Rosa & Esperandio (2022) | 308 participants  G.I.:  138 (44.8%) cis male;  138 (44.8%) cis female;  32 (10.4%) transvestigêneres.  S.O.:  61% Homosexual; 28.2% Bisexual;  6.5% Pan;  1.9% Asexual; 1.6% Heterosexual;  0.6% Questioning. | | 18 to 68 years old (*M* = 28.66 years) | | 20.1% Catholic;  8.8% Evangelical denomination –  2.6% Inclusive Evangelical denominations;  1% Dual membership (Catholic and Spiritist, Catholic  and Kardecist). | Spirituality and Religiosity of sexual and gender minorities, seeking to identify  spiritual resources and the correlation between Religious and Spiritual Struggles and style of attachment to God. | Brazil | Quantitative | Data collection:  Online survey;  Data analysis:  Statistical analysis. | Religious and spiritual struggles (Exline et al., 2014);  Minority Stress Theory (Meyer, 2003).  Complementary theory:  Attachment to God Theory (Kirkpatrick & Shaver, 1992). |
| 77 – Lefevor, Goldblum, et al. (2022) | N.A. | | N.A. | | N.A. | Sexual identity confusion and conflict.  Clinical approaches that have been used  historically to inform treatment with clients experiencing sexual identity confusion and/or conflict.  Ethical and clinical principles. Assessment and treatment recommendations. | USA | Theoretical article | N.A. | Conflict |
| 78 – Puckett et al. (2018) | 217 participants  G.I.:  99 (45.6%)  Men;  118 (54.4%) Women.  S.O.:  168 (77.4%)  Gay or Lesbians or Same-gender loving or Queer;  49 (22.6%)  Bisexuals. | | 18 to 75 years  Old (*M* = 36.36 years) | | 46 (21.2%)  Protestant;  18 (8.3%)  Catholic;  1 (0.5%)  17 (7.8%)  Nondenominational Christian. | How sexual minority participants in the United States viewed religion and their relationships with a higher power, in addition to more descriptive information regarding religious affiliations and attendance at religious services. | USA | Mixed Methods | Data collection:  Online survey with closed and open-ended questions.  Data analysis:  Statistical analysis for quantitative data and content analysis for qualitative data. | Minority Stress Theory (Meyer, 2003) |
| 79 – Longo et al. (2013) | 250 participants  G.I.:  116 (46.4%) females;  115 (46.0%)  males;  19 (7.6% ) transgender  S.O.:  92 (36.8%) gay;  85 (34.0%)  bisexuals or pansexuals;  49 (19.5%) lesbians;  15 (6.0%) questioning;  9 (3.6%) other - e.g., queer, asexual. | | 13 to 25 years old | | 78 (31.2%) Christian faith tradition | The role of religious  tradition and religiosity in engaging in non-suicidal  self-injurious (NSSI) behavior. | USA | Quantitative | Data collection:  Online survey;  Data analysis:  Statistical analysis. | Conflict / “competing selves”(Sherry et al., 2010) |
| 80 – Sowe et al. (2017) | 1600 participants  G.I.:  51.7% female;  48.0% male;  0.3% other.  S.O.:  600  heterosexuals;  716 bisexuals;  284 same-sex attracted – SSA. | | 18 to 75 years  old (*M* = 29.69 years) | | 423 Christian – currently and in the past;  729 Former Christian;  17 converted to Christianity at a later point in life. | Antigay religious exposure and detrimental outcomes across a  variety of clinically relevant measures (e.g., conflict/identity conflict between religious beliefs and sexual minority identity). | USA; | Quantitative | Data collection:  Online survey;  Data analysis:  Statistical analysis. | Conflict |
| 81 – Mosher et al. (2019) | 159 participants  G.I.:  67.3% cis-female;  26.4% cis-male;  1.9% trans-male;  1.3% trans-female;  3.1% other.  S.O.:  63.5% bisexual;  10.7% lesbian; 10.7% gay; 6.3% queer;  8.8% other. | | 18 to 61 years old (*M* = 26.34 years) | | 63.5% Christian. | Perceptions of cultural humility in participants of a  religious group;  The benefits of perceived group cultural humility for LGB populations in a religious context. | USA | Quantitative | Data collection:  Online survey;  Data analysis:  Statistical analysis. | Conflict;  Spiritual/religious struggles;  Minority Stress Theory (Meyer, 2003). |
| 82 – Gerena (2019) | 1 gay man | | N.A. | | Pentecostal Church | Author´s own experiences with religious institutions and the ongoing conflict between religious beliefs and  Sexuality. | USA | Qualitative | Autoethnography | Conflict |
| 83 – Deguara (2018) | 25 participants  G.I.:  16 cis men;  1 trans;  7 cis woman. | | All in the mid-30s | | All catholics | How LGBT Catholics imagine God and how images of God change in parallel with their self-image across the resolving identity conflict process. | Italy | Qualitative | Data collection:  Participant observation; personal in-depth, unstructured interviews.  Data analysis:  Content analysis | Identity conflict/incongruence  Reconciliation |
| 84 – Hall (2015) | 48 participants  G.I.:  27 men;  21 women.  S.O.:  All non-heterosexual | | 19 to 77 years old | | All catholics | The choices faced by Polish non-heterosexual  Christians as a result of their need to integrate their religiosity and sexuality | Poland | Qualitative | Data collection:  Participant observation; Interviews.  Data analysis:  Content analysis | Identity integration |
| 85 – Quinn & Dickson-Gomez (2016) | 30 Young, Black Men who have Sex with Men (YBMSM) | | 16 to 24 years (*M* = 21) | | Christian Black Church | The experiences of  homonegativity among YBMSM with a particular emphasis  on the influence of the Black Church. | USA | Qualitative | Data collection:  Interviews;  Data analysis: Thematic Analysis informed by the principles of Grounded Theory | Stigma (Goffman, 1963) - homonegativity;  Intersectional framework. |
| 86 – Liboro & Walsh (2016) | 9 participants  G.I.:  All male;  S.O.:  All gay. | | 31 to 70 years old | | All Catholics | The phenomenon of HIV-positive gay men in Canada persevering with their Catholic faith  despite the Catholic institution’s known stance against homosexuality and its reputation for  contributing to the stigmatization of HIV/AIDS. | Canada | Qualitative | Data collection:  Interviews;  Data analysis: Grounded Theory | Identity conflict termed in this article as “identity incongruity” (Liboro, 2004).  Complementary theories:  Kohlberg’s (1981) stages of moral development;  Fowler’s (1981) stages of faith development. |
| 87 – Stamatoulakis & Nearchou (2015) | 1 participant  G.I.: girl;  S.O.: lesbian. | | 20 years old | | Christian Protestant Church of Norway | The conflict between faith and sexual identity in  female homosexual priests-to-be. | Norway | Qualitative  (case study) | Data collection:  Interview;  Data analysis: IPA | Conflict |
| 88 – Hamblin & Gross (2013) | 193 participants  G.I.:  124 men (64.2%);  29 women (35.8%). | | 21 to 86 years old (*M* = 49 years) | | 63% Protestant;  23% Catholic;  Jewish (6%)  3% Other Christian affiliations. | Frequency and type of church attended and  psychological well-being in homosexual population, including measures of social support, identity conflict, anxiety, and  depression. | USA | Quantitative | Data collection:  Online survey;  Data analysis:  Statistical analysis. | Identity Conflict (Rodriguez & Ouellette, 2000) |
| 89 – Schindler (2021) | 105 participants  G.I.:  27 Male;  65 Female.  S.O.: All LGB. | | 18 to 65+ years old | | Christian not specified; Catholic; Mormon. | The relationship between religious stress in childhood and substance use disorders in adulthood among sexual minorities. | USA | Quantitative | Data collection:  Online survey;  Data analysis:  Statistical analysis. | Religious stress (Page et al., 2013) |
| 90 – Rabasco & Andover (2021) | 180 participants  G.I.:  99 (55%) Trans women;  33 (18.3%) Trans men;  9 (5%) Gender non-conforming;  9 (5%) Gender queer;  30 (16.7%) Other).  S.O.:  38 (21.1%) Bisexual;  34 (18.9%) Pansexual;  27 (15%) Lesbian;  24 (13.3%) Heterosexual;  19 (10.6%) Queer;  14 (7.8%) Gay;  6 (7.7%) Other;  10 (5.6%) Asexual. | | 18 to 55 years old  (*M* = 26.01 years) | | 21 (10.5%) Christian denomination  . | How different aspects of one’s religion, including formal religious practices, belief in God, and religious conflict, are associated with suicidal ideation severity and  lifetime frequency of suicidal behaviors among a sample of TGD  adults. | USA (mostly); Canada; United Kingdom. | Quantitative | Data collection:  Online survey;  Data analysis:  Statistical analysis. | Identity Conflict (with the same measurement as J. J. Gibbs & Goldbach, 2015) |
| 91 – Craig et al. (2017) | 40 participants  G.I.:  All girls;  S.O.:  Lesbian (*n* = 15);  Bisexual (*n* = 20);  Questioning (*n* = 4);  Queer (*n* = 1). | | 15 to 18 years old (*M* = 16 years) | | All Christian Catholics | Experiences of  stress and resilience among Etnoracial Sexual Minority Girls (ESMG) | USA | Qualitative | Data collection:  Focus groups;  Data analysis: Grounded Theory | Minority Stress Theory (Meyer, 2003);  Intersectionality;  Resilience;  Conflict. |
| 92 – Dean et al. (2021) | 160 participants  G.I.:  72 (45%) female;  81 (51%) male;  4% Other (2 transgender, 2  genderfluid,  1 genderqueer,  1 agender,  1 unknown).  S.O.:  80 (50%) Publicly heterosexual;  9 (5.6%)  Privately heteresexual;  75 (46.9%) privately LGB. | | *M* = 21.4 years old | | All Christians (with no identified Christian affiliation). | The relationship between faith and sexual identity and its implications for psychological  health within the Christian college environment. | USA (mostly); Canada; United Kingdom. | Quantitative | Data collection:  Online survey;  Data analysis:  Statistical analysis. | Conflict;  Psychological distress. |
| 93 - Kralovec et al. (2014) | 358 participants (the LGB group)  G.I.:  114 (32%) women;  244 (68%) men. | | *M* = 36.41 years old | | 183 (51%) Catholic;  29 (8%) Protestant. | The effect of religion on suicide risk | Austria | Quantitative | Data collection:  Online survey;  Data analysis:  Statistical analysis. | Identity Conflict |
| 94 - Rosik et al. (2022) | 1317 participants  G.I.:  955 (72.5%) male;  293 (22.2%) female;  14 (1.1%) transwoman;  11 (0.8%) more male than female;  11 (0.8%) gender fluid;  9 (0.7%) more female than male;  9 (0.7%) gender queer;  15 (1.1%) others.  S.O.:  455 (34.5%) lesbian or gay;  212 (15%) SSA;  115 (8.7%) heterosexual with SSA;  85 (6.5%) heterosexual/straight;  70 (5.3%) bisexual;  90 (6.8%) other. | | 18 to 80 years old  (*M* = 38.91 years) | | 44 (3.3%) Catholic;  39 (3%) Evangelical protestant;  776 (77.6%) LDS;  15 (1.1%) Baptist. | The association between conservative religiousness inherently and poorer health in sexual minorities. | USA | Quantitative | Data collection:  Online survey;  Data analysis:  Statistical analysis. | Minority stress theory (Meyer, 2003)  Conflict / identity conflict |
| 95 - Baiocco et al. (2014) | 544 participants  G.I:  316 Men;  228 Women.  S.O:  316 Gay;  228 Lesbian. | | 18 to 35 years  old  (*M* = 26.52 years) | | All currently Catholics or raised in catholic context | Evaluate in  lesbian and gay young adults the role of internalized sexual stigma on the development  of a repulsion by life attitude as a potential predictive factor of  suicidal ideation. | Spain;  Italy. | Quantitative | Data collection:  Online survey;  Data analysis:  Statistical analysis | Minority stress theory (Meyer, 2003) |
| 96 - Best & Weerakoon (2021) | 30 participants  G.I.:  11 Biological sex as female;  8 Trans man;  8 Trans woman;  7 Biological sex as male with “gender dysphoria”;  2 Biological sex as female with “gender dysphoria”;  1 De-transitioned biological male;  1 De-transitioned biological female;  2 Not clearly stated. | | 18 to 78  years old  (M = 48.2 years) | | All Christians | The interaction between Christian faith and gender incongruence. | Australia;  USA;  UK. | Qualitative | Data collection:  Survey with open-ended questions;  Data analysis: Grounded Theory | Identity conflict / tension |
| 97 - Dangerfield II. et al. (2019) | 26 participants  G.I.:  All men.  S.O.:  69% Gay;  31% Bisexual. | | 24 to 61 years old | | All participants currently affiliated or raised in Christian Black Church. | Personal meanings of religiosity and spirituality and how these constructs afect partner choices and sexual behaviors among Black Gay and Bisexual Men. | USA | Qualitative | Data collection:  Interviews  Data analysis:  Content analysis | Cognitive Dissonance;  Intersectional framework. |
| 98 - Heiden-Rootes et al. (2021) | 384 participants  G.I.:  138 Male;  203 Female; 38 Non-binary/Transgender.  S.O.:  153 Bi+;  115 Gay;  116 Lesbian. | | Bi+  (*M* = 29.67 years);  Gay (*M* = 36.94 years); Lesbian  (*M* = 34.22 years). | | 30 Catholic;  38 Protestant ;  33 LDS;  68 Other Christian. | Religiously based minority stressors for those raised in religious families that interacted with religious coping to predict mental health. | USA | Quantitative | Data collection:  Online survey;  Data analysis:  Statistical analysis | Minority stress theory (Meyer, 2003) |
| 99 - Barajas (2014) | N.A. | | N.A. | | N.A. | Intersectionality as a starting point with respect to religion and gender, for through the main concepts of bourdian theory give an account of the processes of exclusion/inclusion and standardization of LGBT identities in the religious field. | Mexico | Theoretical article | N.A. | Intersectionality theory (Crenshaw, 1991)  Complementary theory:  Bourdieu´s (1995, 2006) theory |
| 100 - Ho & Hu (2016) | 28 participants  G.I.:  2 transgender  Note: There is no more information regarding participants gender identity.  S.O.:  13 gay;  8 Lesbian;  4 Bisexual. | | 16 to 50 years old | | 26 Protestants;  1 Ex-Protestant;  1 Catholic. | How the sexual self of sexual minorities has been marginalized and excluded in intimate social spaces of family, church communities and schools in Hong Kong with specific spatial practices and different forms of power/knowledge. | Hong Kong | Qualitative | Data collection:  Interviews;  Focus groups.  Data analysis:  Thematic analysis | Identity conflict;  Feminist geography.  Complementary construct:  “personal life” from Smart (2007) |
| 101 - Grigoriou (2014) | 142 participants  G.I.:  106 (75%) of male;  36 (25%) female.  S.O.:  Gay;  Lesbian; Same-sex attracted; Same-gender attracted; Homosexual; Queer; Gender-bending;  Butch;  Dyke;  Bisexual.  Note: There is no statistical information regarding each self-identified sexual orientation/identity. | | *M* = 40.37 years old | | All Mormon | The intersection of sexual identity, religious identity, perceptions of stigmatization, and social constraints on a sample of adults raised from birth in Mormon society. | USA | Quantitative | Data collection:  Online survey;  Data analysis:  Statistical analysis | Minority stress theory (Meyer, 2003) |
| 102 - Gabriele-Black & Goldberg (2021)  aparece 107 no Covidence | 23 participants  G.I.:  15 (65.2%) Cisgender;  6 (26.1%)Trans 4 (17.4%) Nonbinary;  1 (4.3%) Gender nonconformin; 1 (4.3%) Feminine-of-center.  S.O.:  7 (30.4%) Gay;  7 (30.4%) Queer;  6 (26.1%) Bisexual;  5 (21.7%) Asexual;  5 (21.7%) Lesbian;  2 (8.7%) Pansexual;  1 (4.3%) Demisexual. | | 18 to 29 years old (*M* = 23.48 years) | | All Evangelicals | How queer emerging adults, who may be in the process of (re)defining their faith (Arnett & Jensen, 2002) based on their emergent sexual/ gender minority identity, advocate (or choose not to advocate) for LGBTQ rights and the ways in which they “queer” what it means to be LGBTQ on an Evangelical Christian campus. | USA | Qualitative | Data collection:  Interviews  Data analysis:  Thematic analysis | Queer theory;  Intersectionality theory. |
| 103 - Gattis et al. (2014)  **Note:** This study has a control group with heterosexual participants. For this analysis, we account the statistics regarding the sexual minority group, our research interest. | 393 sexual minority participants  G.I.:  67% Female;  33% Male.  S.O.:  12% Completely gay/lesbian;  8 % Mostly gay/lesbian;  17 % Bisexual;  63 % Mostly heterosexual. | | *M* = 23.4 yeras old | | 16% Churches that endorsed same-sex marriaged;  19% Churches that opposed same-sex marriage;  5% Churches with an undefined position on same-sex marriage. | The role of religious affiliation as a protective factor on the relationship between perceived interpersonal discrimination and depression. | USA | Quantitative | Data collection:  Online survey;  Data analysis:  Statistical analysis | Risk and protective factor framework |
| 104 - Lefevor et al. (2020) | 1123 participants  G.I.:  67.1% Woman;  30.4% Man; 1% Transgender;  1.5% Self-identify.  S.O.:  4.4% Straight; 18.6% Lesbian;  16.1% Gay;  28.1% Bisexual;  18.1% Questioning;  14.7% Self-identify. | | *M* = 22.13 years old | | 54.2% Christian not specified;  3.1% Catholic.  . | The prevalence of various distal stressors (i.e., objective experiences of discrimination and/or oppression that do not depend on an individual’s perception) and forms of support (e.g. family, partner, and religious support) and their relationship with psychological distress among Black sexual minorities. | USA | Quantitative | Data collection:  Online survey;  Data analysis:  Statistical analysis | Intersectionality theory (Crenshaw, 1989; Cole, 2009)  Resilience. |
| 105 - Myler (2013) | 24 participants  Intervention group 1 (*n* = 5)  G.I.:  All male;  S.O.:  Lesbian/gay (*n* = 4); Questioning (*n* = 1).  Group 2 (*n* = 7)  G.I.:  Male (*n* = 6);  Female (*n* = 1).  S.O.:  Lesbian/gay (*n* = 6); Questioning (*n* = 1). | | Group 1:  *M* = 29 years old  Group 2:  *M* = 26 yeras old | | - Group 1  All LDS (*n* = 5); 6  - Group 2  LDS (*n* = 6);  Other (*n* = 1). | The clinical effectiveness of an Acceptance and  Commitment Therapy (ACT) group intervention for individuals reporting distress related  to conflict between sexual and religious identity. | USA | Quantitative | Data collection:  Online survey;  Data analysis:  Statistical analysis | Conflict |
| 106 - Zarzycka et al. (2017) | 108 participants (divided into two groups - one intervention group with homosexual participants and one control group with heterosexual participants)  Note: We extracted data regarding the intervention group (G1).  G1 (*n* = 54)  G.I.:  All men;  S.O.:  All homosexuals. | | Group 1: *M* = 26.40 (SD = 5.59) | | All Roman Catholic | The analysis of relationships between religious comfort and struggle with state anxiety and satisfaction with life in homosexual man. | Poland | Quantitative | Data collection:  paper survey  Data analysis:  Statistical analysis | Religious struggles;  Conflict. |
| 107 - Chestna (2015) | 4 particpants  G.I.:  All women;  S.O.:  All lesbians. | | 18 to 22 years old | | All Roman Catholic | The experiences of undergraduate Catholic lesbians as they negotiate the potentially dissonant religious and sexual aspects of their identities. | USA | Qualitative | Data collection:  Interviews;  Data analysis:  Interpretative phenomenological analysis | Conflict;  Cognitive Dissonance |
| 108 - Hill (2015) | 10 participants  G.I.:  4 Women;  4 Men;  2 Transgender.  S.O.:  4 Gay;  3 Lesbians;  2 Heterosexual (the transgender participants);  1 Bisexual.  **Note:** One transgender participant was excluded because the interview recording was corrupted. | | 26 to 57 years old | | Previous religion affiliation:  6 Baptist;  1 Christian;  1 Catholic;  1 Jehovah’s Witness;  1 African Methodist and Episcopal Zion. | The  lived experience of Black LGBT individuals with respect to spirituality and how they  experience and cope with homonegativity within traditional organized religions. | USA | Qualitative | Data collection:  Interviews;  Data analysis:  Phenomenological analysis | Pargament’s (1997) theory on the psychology of religion and coping;  Feminist Theory. |
| 109 - Mundell (2016) | 8 participants  G.I.:  All men;  S.O.:  All gay. | | 24 to 78 years old | | All current or former Jehovah’s  Witnesses (JW). | Experiences of psychotherapy interventions that JW  gay men, whose core religious and sexual orientations are at significant conflict with each other,  found most helpful in the client’s efforts at integrating these two aspects of their identities. | USA | Qualitative  (specifically the Critical Incident Technique (CIT). | Data collection:  Interviews;  Data analysis:  According to the fourth step of CIT. | Conflict:  Integration/Reconciliation |
| 110 - Klundt et al. (2020) | 7625 participants  Sex at birth:  4235 female-at-birth (68%);  3295 male-at-birth (43%);  94 not identify sex-at-birth (1%).  S.O.:  86.8% exclusively hererosexual;  4.5% predominantly heterosexual, only incidentally homosexual;  2.1% predominantly heterosexual, but more than incidentally homosexual;  1.5% equally heterosexual and homosexual;  1.2% predominantly homosexual, but more than incidentally hererosexual;  0.8% predominantly homosexual, only incidentally hererosexual;  1.8% exclusively homosexual. | | 18 to 65 years old | | 95% LDS. | The religiosity, mental health outcomes, and sexual minority identity at a highly religious university. | USA | Quantitative | Data collection:  online survey  Data analysis:  Statistical analysis | Minority stress theory |
| 111- Thamrin et al. (2022) | 337 participants  G.I.:  74.3% cisgender male;  18.6 cisgender female;  6.4% transgender.  S.O.:  84% gay/lesbian  7% bisexual  9% queer/other | | 14 to 24 years old  (*M*  =  20.29 years) | | 38% Catholic;  34% Christian not specified. | The analysis of discrimination and depressive symptoms in sexual minorities moderated by religious importance and attendance. | USA | Quantitative | Data collection:  online survey  Data analysis:  Statistical analysis | Risk and resilience framework (Luthar et al.,  2000) |
| 112 - Mascaro (2017) | 10 participants  G.I.:  All men;  S.O.:  All gay. | | 46 to 66 years old | | Roman Catholic | The experience of psychic  dissonance on gay diocesan priests in active ministry within the Catholic Church while at  the same time coming to terms with their own understanding and acceptance of gay  identity. | USA | Qualitative | Data collection:  Interviews;  Data analysis:  Phenomenological analysis | Cognitive Dissonance;  Conflict. |
| 113 - Sowe et al. (2014) | 579 participants  G.I.:  51.6% male;  46.8% female;  2.4% other.  S.O.:  45.8% gay;  26.1% lesbian;  17.1% bisexual;  8.6% other (e.g., queer);  2.4% straight. | | 18 to 74 years old (*M* = 31.76 years) | | All Christians or former Christians | Comparing religious and nonreligious same-sex attracted adults on internalized homonegativity and distress. | Australia | Quantitative | Data collection:  online survey  Data analysis:  Statistical analysis | Conflict |
| 114 - Hibma (2018) | 4 participants  G.I.:  3 male;  1 female.  S.O.:  3 gay;  1 sexual minority (without a specific sexual minority identity label). | | 21 to 27 years old | | All Christian Evangelical | How students at Christian evangelical colleges developed their religious and sexual  identities and how these two areas interacted. | USA | Qualitative | Data collection:  Interviews;  Data analysis:  Phenomenological analysis | Conflict;  Meyer’s (2003) minority stress model.  Complementary theory:  Fowler’s (1981)  faith development theory.  . |
| 115 - Grimes (2020) | 21 participants  G.I.:  All transgender women.  S.O.:  Without statistical information regarding participants' sexual orientation. | | 25 to 63 years old  (*M* = 46.95 years old) | | Diverse  Christian denominations  (African Methodist Episcopal, Baptist,Pentecostal, and SDA). | The perceptions that self-  identified transgender women of color living with HIV possessed regarding  spirituality, religion, and HIV care. | USA | Qualitative | Data collection:  Interviews;  Data analysis:  Grounded Theory. | Intersectionality;  Empowerment Feminist Theory.  Complementary theory:  Andersen’s (1995) Behavioral Model. |
| 116 - Hollier et al. (2022) | 24 participants  G.I.:  11 men;  10 women;  1 transgender man;  1 transgender woman;  1 non-binary.  S.O.:  10 gay;  7 lesbians;  3 bisexuals;  1 asexual. | | Missing | | All Christian Evangelical | A trauma-informed lens to explore the  ordeal that generally accompanies being LGBTQIA + in  Evangelical churches. | Australia | Qualitative | Data collection:  Interviews;  Data analysis:  Critical Realist framework | Religious Trauma    Minority Stress and  Microaggression as two mechanisms behind religious trauma. |
| 117 - Paul (2019) | 655 participants  G.I.:  54.8% male; 29.5% female;  7% transgender;  6.9% genderqueer/fluid;  1.8% other gender.  S.O.:  63.2% lesbian or gay;  16.9% bisexual; 12.2% queer; 0.8% heterosexual. | | 18 to 82 years old  (*M* = 35.03 years) | | 47.6% Christian;  3.1% Buddhist;  2.7% Hindu; 3.7% Jewish; 1.1% Muslim; 15.1% agnostic;  11.8% atheist; 15.0% other. | Explore the intersection between sexual minority and religious/spiritual experiences through the relationships between sexual minority  identity development, religious schemata, R/S struggles, and life satisfaction. | USA | Quantitative | Data collection:  Online survey  Data analysis:  Statistical analysis | Identity integration;  Religious/Spiritual struggles.  Complementary theory:  Streib’s (2001) religious styles model.  . |
| 118 - Christian (2016) | 346 participants (quantitative);  G.I.:  All females;  S.O.:  83% Straight;  3% Gay / lesbian / homosexual;  11% Bisexual;  2% Asexual;  1% Not sure.  8 participants (qualitative)  G.I.:  All females;  S.O.:  50% Straight;  50% Bisexual. | | 18 to 25 years old  (Quanti study: *M* = 21 years; Quali study: *M* = 19.5 years ) | | Quantitative phase:  21% Catholic;  1% Evangelical Protestant;  4% Protestant;  12% Baptist;  1% Unspecified Christian;  1% Mormon;  Qualitative phase:  25% Catholic;  4% Unspecified Christian. | The experience of loneliness related to  religious and spiritual struggles for female college students. | USA | Mixed methods | Data collection:  Online survey;  Interviews.  Data analysis:  Statistical analysis;  Thematic analysis. | Religious/Spiritual struggles |
| 119 - Black (2018) | 29 participants  G.I.:  12 (13.0%) cis-women;  7 (24.1%) cis-men;  3 (10.3%) non-binary;  3 (10.3%)  trans men;  1 (3.4%) non-binary/trans;  1 (3.4%)  feminine of center;  1 (3.4%) femandrogynous;  1 (3.4%) gender  nonconforming/transgender/a transman.  S.O.:  7 (24.1%) gay;  5 (17.2%) queer;  4 (13.8%) lesbian;  3 (10.3%) bisexual;  2 (6.9%) asexual;  2 (6.9%) bisexual/asexual;  1 (3.4%) pansexual;  1 (3.4%) queer/asexua;  1 (3.4%) gay/lesbian/queer;  1 (3.4%)  gay/lesbian;  1 (3.4%) lesbian/queer/demisexual; 1 (3.4%) bisexual/queer/pansexual. | | 18 to 29 years old (*M* = 24.07 years) | | All participants had attended at least one Evangelical church during their  childhood and adolescence. | The ways in which queer emerging adults from Evangelical backgrounds  resist, transform, or modulate Evangelical discourse while navigating identity  challenges unique to their social locations. | USA | Mixed-methods | Data collection:  1st phase (qualitative) - Interviews;  2nd phase (quantitative) - Online survey;  Data analysis:  Thematic analysis; Statistical analysis.  . | Queer theory;  Tension;  Integration.  Complementary theory:  Arnett´s (2000) emerging adulthood identity development model. |
| 120 - Sadusky (2018) | 14 participants  G.I.:  8 men;  6 women.  S.O.:  7 gay;  4 same-sex attracted;  1 bisexual;  2 do not use a sexual identity label. | | 18 to  75 years old | | 7 Roman Catholic;  3 Various Christian denominations.. | The experience of loneliness and coping with loneliness for people navigating questions of sexual and religious identity. | USA | Qualitative | Data collection:  Interviews;  Data analysis:  Consensual Qualitative Research | Conflict;  Cognitive dissonance.  Complementary theory:  Yarhouse & Tan´s (2004) Sexual Identity Development. |
| 121 - Coley (2020) | 65 participants  G.I.:  54% men;  8% transgender or gender fluid.  S.O.:  77% lesbian, gay, or bisexual. | | No information | | All Christians | How LGBTQ activist groups shape participants’ approaches to understanding the connections between religion and sexuality. | USA | Qualitative | Data collection:  Interviews;  Data analysis:  Content analysis | Conflict |
| 122 - Lefevor et al. (2020) | 1128 participants  - LGBQ  (*n* = 724)  G.I.:  24.6% Woman;  67.7% Man;  2.5% Transgender;  5.2% Gender non-conforming.  - SSA (*n* = 404)  G.I.:  22.8% Woman;  73% Man;  2.2% Transgender;  2% Gender non-conforming. | | *M* = 36.12 years old | | All Mormon or ex-mormon. | The relationship between rejecting an LGBQ  identity and religiousness, attitudes toward sexuality, and  health outcomes. | USA | Quantitative | Data collection:  Online survey  Data analysis:  Statistical analysis | Intersectionality  Minority Stress Theory |
| 123 - Crocker (2022) | 10 participants  G.I.:  70% men;  30% woman  S.O.:  50% gay;  20% lesbian;  10% bisexual;  10% queer;  10% same-sex attracted | | 21 to 41 years old | | All Christians | The exploration of Religious Trauma and Spiritual Resilience in  Sexual Minority Christians. | USA | Qualitative | Data collection:  Interviews;  Data analysis:  Consensual Qualitative Research | Conflict and cognitive dissonance  Minority stress theory  Religious trauma  (Spiritual) Resilience |
| 124 - Jacobsen (2013) | 23 participants  G.I.:  All woman;  S.O.:  19 lesbians;  2 bisexuals;  1 heterosexual;  1 Same-sex attracted. | | 20 to 56 years old;  *M* = 37 years old. | | 11 Mormon | The phenomenon of experiencing conflict between central aspects to core concept of Self, i.e., spirituality and sexuality. | USA | Qualitative | Data collection:  Interviews;  Data analysis:  Thematic Analysis | Intersectionality framework;  Conflict.  . |
| 125 - Radojcic (2016) | Two groups:  a) Dignity (15 participants; all gay men; with regular presidents lesbian women);  b) Log Cabin Republicans (without number; all gay men, with one female visitor occasionally) | | a) most over 65 years old;  b) one group with 65 years and older; another group between 30 and 40 years old. | | Dignity (all catholics);  Log Cabin Republicans (no information regarding religious affiliation). | The experiences of  LGBT people who participate in organizations that are pushing for the acceptance of  sexual minorities in the Catholic Church and the Republican Party. | USA | Qualitative | Data collection:  Participant observation;  Interviews;  Analysis of archival materials.  Data analysis:  Content Analysis | Conflict and conflict resolution. |
| 126 - Claybaugh (2014) | 113 participants  G.I.: All man  S.O.: All gay | | 18 to  76 years old (*M* = 40.85; SD= 13.39) | | Religion growing up:  36 (31.9%)  Catholic;  45 (39.8%)  Protestant;  18 (15.9%)  Mormon;  Religion Current:  20 (17.7%)  Catholic;  18 (15.9%)  Protestant;  9 (8%)  Mormon. | The relationship between level of religiosity and  past suicidal ideation in gay males. | USA | Quantitative | Data collection:  Online survey;  Data analysis:  Statistical analysis | Conflict  Complementary theory:  Pescosolido & Georgianna´s (1989) network theory of suicide |
| 127 - Skidmore (2017) | 8 participants:  G.I.:  5 males (one transgender male);  3 females.  S.O.:  3 Gay;  2 Lesbians;  2 Bisexuals;  1 No information. | | 18 to 23 years old | | 4 Catholic;  3 Baptist. | Collegiate experiences as LGBT and identifying  with an organized religion. | USA | Qualitative | Data collection:  Interviews;  Data analysis:  Thematic Analysis | Queer theory;  Intersectionality theory |
| 128 - Barnes & Meyer (2012) | 355 participants  G.I.:  178 women;  177 men.  S.O.:  All lesbian, gay or bisexual. | | 18 to 58 years old (*M* = 32.6 years) | | 71 Catholic;  45 Protestant. | Affiliation with non-affirming religious settings and internalized homophobia. | USA | Quantitative | Data collection:  Online survey;  Data analysis:  Statistical analysis | Minority Stress Theory (Meyer, 2003) |
| 129 - Harvey & Ricard (2018) | N.A. | | N.A. | | N.A. | The benefits of using intersectionality as a context for exploring intersecting identity experiences by highlighting layered identities of African American women and gay men who have experienced sexism and heterosexism within the Black Church. | USA | Theoretical article | N.A. | Intersectionality |
| 130 - Rosik et al. (2021) | 274 participants  G.I.:  62 women;  209 men;  14 other descriptions (e.g., transman, gender fluid, genderqueer).  S.O.:  96 (35%) LG;  41 (15%) SSA;  30 (10.9%) Heterosexual with SSA;  13 (4.7%) Bisexual;  13 (4.7%) Homosexual;  12 (4.4%) Straight ;  12 (4.4%) Not Use a Label;  8 (2.9%) Queer;  26 (9.5%) Other;  23 (8.4%) No Option/  More than One Applies. | | *M* = 42.11 years old | | 40 (14.6%) Catholic;  32 (11.7%) Evangelical Protestant;  15 (5.5%)  Baptist;  12 (4.4%) Jehovah’s Witness;  11 (4%) Methodist;  11 (4%) Pentecostal. | Characteristics of sexual minorities who reject an LGB identity/identification and how these characteristics may distinguish  them from sexual minorities who are LGB-identified. | USA | Quantitative | Data collection:  Online survey;  Data analysis:  Statistical analysis | Minority Stress Theory (Meyer, 2003) |
| 131 - Lefevor et al. (2022) | 260 participants  G.I.:  46% Cisgender men;  30% Cisgender women;  24% Transgender or non-binary.  S.O.:  58% Bisexual/pansexual;  30% Gay/lesbian;  5% SSA;  3% No label;  5% Other. | | *M* = 33.1 years old. | | 27% Christian not specified; 15% Catholic. | From minority stress theory (Meyer, 2003) and intersectionality theory (Collins, 1989; Crenshaw, 1989) to situate the role that internalized stigma may play for individuals with minoritized sexualities who hold a religious identity. | USA | Quantitative | Data collection:  Online survey;  Data analysis:  Statistical analysis | Minority Stress Theory (Meyer, 2003);  Intersectionality (Crenshaw, 1989). |
| 132 - Hedge (2017) | 6 participants  G.I.:  All men;  S.O.:  All SSA; One of them identified as gay. | | 40 to 55 years old | | All evangelical | The experiences of  SSA men from a conservative/evangelical church background with  competing spiritual and sexual identities. | USA | Qualitative | Data collection:  Interviews;  Photo-elicitation.  Data analysis:  Pattern matching and explanation building. | Conflict |
| 133 - Goodrich et al. (2016) | 12 participants  G.I.:  8 females;  4 males.  S.O.:  4 gay;  6 lesbians;  2 bisexuals. | | 18 to 58 years | | 9 Christian Lutheran, Mormon,  Presbyterian, Roman Catholic. | The meaning  and experiences of counseling for LGB individuals, specifically  the ways in which counseling addressed their sexual and  religious/spiritual identities. | USA | Qualitative | Data collection:  Interviews;  Data analysis:  Consensual Qualitative Research | Conflict/Tension  Identity integration/Resolution |
| 134 - Hanlon (2013) | 4 participants  G.I.:  All women;  S.O.:  All lesbians. | | 31 to 51 years old  (*M* = 45.5 years old) | | All catholics | How lesbian Catholics remain in the Church, and their ways of defending and coping with conflict.  The influence of the Church on the self-experience of gay women who  continue to practice as Catholics. | USA | Qualitative | Data collection:  Interviews;  Data analysis: | Conflict and integration;  Complementary theory:  Self-psychology theory  (Kohut, 1971) |
| 135 - McKinney & Storlie (2021) | 8 participants  G.I.:  All men;  S.O.:  All gay. | | 27 to 72 years old  (*M* = 47 years old) | | 3 United Church of Christ;  2 Catholic;  2 Episcopalian;  1 Unitarian Universalist. | The stories of individuals  holding a Christian religious identity and gay sexual identity. | USA | Qualitative | Data collection:  Interviews;  Data analysis:  Narrative analysis | Conflict and Identity integration |
| 136 - Szymanski & Carretta (2020) | 193 participants  G.I.:  49% Woman;  37% Man;  8%Genderqueer/gender non-conforming;  3% Transman;  3% Transwoman.  S.O.:  71% Lesbian/Gay;  29% Bisexual. | | 18 to 75 years old  (*M* = 37.73 years old) | | 69% Christian | The relations between religious sexual stigma and both psychological distress and  wellbeing among LGB persons who were affiliated with a religion/faith via  a moderated mediation model. | USA | Quantitative | Data collection:  Online survey;  Data analysis:  Statistical analysis | Religious struggle |
| 137 - Dehlin et al. (2015) | 1493 participants  G.I.:  1,138 (76%) men;  355 (24%) women.  S.O.:  All SSA | | *M* = 36.8 years old | | All current or former members of the LDS. | The many ways in which SSA-LDS adults  manage their identity conflict. | USA and 21 other countries (not specified). | Quantitative | Data collection:  Online survey;  Data analysis:  Statistical analysis | Identity Conflict  Cognitive Dissonance Theory |
| 138 - Trecartin et al. (2022) | 299 participants  G.I.:  135 (44.4%) Male;  132 (43.4%) Female;  8 (2.6%) Transgender;  29 (9.6%) Other.  S.O.:  63 (20.7%) Lesbian;  115 (37.8%) Gay;  87 (28.6%) Bisexua ;  39 (12.8%)  Other. | | 18 to 35 years old | | All raised Seventh Day Adventist | The experiences of LGBTQ+ millennials who were raised in the Seventh-day Adventist Church, namely the role of religiosity and substance use as risk and protective factors for depression  and suicidality. | USA | Quantitative | Data collection:  Online survey;  Data analysis:  Statistical analysis | Minority Stress Theory |
| 139 - Fallon et al. (2013) | N.A. | | N.A. | | N.A. | Critical thinking to assist counselors to integrate religious and spiritual beliefs with  professional ethical codes. | USA | Theoretical Article | N.A. | Conflict |
| 140 - McGuire et al. (2017) | 21 participants  G.I.:  16 Women;  5 Men.  S.O.:  Not specified, but most participants discuss being a sexual minority (e.g., gay, queer) in the analysis excerpts. | |  | | 16 Christian not specified;  1 Catholic;  1 Secular  with Christian households. | Students' spiritual, race, and gendered identities in interaction with their sexual identities. | USA | Qualitative | Data collection:  Interviews;  Data analysis:  Thematic analysis | Black feminism;  Queer of Color Critique. |
| 141 - Garrett-Walker & Torres (2017) | 20 participants  G.I.:  All men;  S.O.:  14 Gay;  3 Queer;  2 Bisexual;  1 Pansexual. | | 18 to 25 years old. | | 9 Christian not specified. | The ways in which religious institutions pontificate  about same-sex behavior and the influence that such rhetoric has on the lives  of Black queer emerging adult men. | USA | Qualitative | Data collection:  Interviews;  Data analysis:  Thematic analysis | Intersectionality |
| 142 - Carrico et al. (2017) | 1565 participants  G.I.:  All men;  S.O.:  All men who have sex with men (MSM); 74% identified as gay. | | 18 to 29 years old (86% with 21 years old). | | All with some link to the Black Church. | Spirituality/religiosity as correlate of binge drinking, stimulant use, and recent HIV testing, | USA | Quantitative | Data collection:  Online survey;  Data analysis:  Statistical analysis | Stress and Coping Theory  (Park and Folkman, 1997) |
| 143 - Crowell et al. (2015) | 634 participants  G.I.:  473 (72%) male;  161 (24.5%) female;  24 (3.5%) transsexual, gender queer,  two-spirit.  S.O.:  65% gay;  14% lesbian;  19% bisexual/sexually fluid;  2% no response. | | 18 to 33 years old (*M* = 26.41 years old). | | 50% LDS;  5% Other Christian denominations.. | Specific aspects of minority stress as  they relate to depression and also differences  based on demographic characteristics. | USA and 22 additional countries worldwide (not specified). | Quantitative | Data collection:  Online survey;  Data analysis:  Statistical analysis | Minority Stress Theory (Meyer, 2003) |
| 144 - Etengoff (2017) | 25 participants  16 gay men;  9 family allies. | | Christian gay men (*M* = 25 years old) | | 14 Christian | How petition letters can help gay men  and their religious family members mediate community conflicts regarding  religion and sexual orientation. | USA | Qualitative | Data collection:  Letter-writing task;  Data analysis:  Narrative analysis | Conflict (external conflict with the religious community in general and/or religious leaders in particular);  Capabilities approach;  Cultural historical activity theory. |
| 145 - Navarrete (2020) | 7 participants  All gay men | | 27 to 40 years old (*M* = 33.14 years old) | | All currently self-identified as catholics or raised catholics. | Intersectional experiences  related to elements of Mexican culture (i.e., machismo, caballerismo, familismo, respeto, personalismo, simpatia, and Catholicism) and gay identity. | USA | Qualitative | Data collection:  Interviews;  Data analysis:  Thematic analysis | Intersectionality  Complementary theory:  Morales´ (1989) identity model |
| 146 - Etengoff & Rodriguez (2017) | 38 participants  23 gay men;  15 family allies (without inforfation regarding their sexual orientation and gender identity). | | Gay men participants: 18 to 34 years old.  Family allies: *M* = 25 years old. | | 21 Christian: Mormon 7 gay men and 4 family allies); Catholic (2 gay men and 1 family ally); Methodist (2 gay men and 1 family ally); Evangelical (1 gay men and 1 family ally); SDA (1 gay men and 1 family ally). | Gay men´s and their family allies scriptural interpretations.  **Note:** In this study, scriptural interpretation can be understood to serve as an agentive avenue for indivuduals´mediation of sociocultural conflicts. | USA | Qualitative | Data collection:  Interviews;  Data analysis:  Thematic analysis | Conflict |
| 147 - Yarhouse et al. (2017) | 8 participants  G.I.:  6 male;  2 without information.  S.O.:  All gay | |  | | All Christians: 6 Protestant Christian;  2 Catholic. | The narratives of celibate gay Christians. | USA | Qualitative | Data collection:  Interviews;  Data analysis:  Grounded theory and Consensual Qualitative Analysis. | Conflict and conflict resolution throughout celibacy. |
| 148 - Lesher (2018) | 1 participant  Intersex, transgender woman | | 42 years old | | United Church of Christ | The experience of being a  transgender, intersex woman, and how her gender identity  intersects with her faith and vocational calling. | USA | Qualitative | Data collection:  Interview;  Data analysis:  Grounded theory | Intersectionality  Complementary theory:  The multidimensional identity model (Reynolds & Pope, 1991) |
| 149 - Watkins et al. (2016) | 1154 participants  G.I.:  All men (who have sex with men);  S.O.:  114 Heterosexual (10%);  739 Homosexual (65%);  255 Bisexual (22.5%);  29 Other (2.5%). | | 18 to 71 years old  (*M* = 41.5 years old). | | All with some link with Black Church. | Religiosity and spirituality associations with high-risk behavior and  high-risk sexual behavior among Black MSM. | USA | Quantitative | Data collection:  Online survey;  Data analysis:  Statistical analysis | Cognitive Dissonance/ Conflict |
| 150 - Reygan & Moane (2014) | 10 participants  G.I.:  6 male;  4 female.  S.O.:  7 gay;  2 bissexual;  1 without label. | | 20 to 50 years old | | 3 Catholic;  1 Anglican. | Experiences of religious homophobia among LGBT people in Ireland given the historical dominance of the Catholic Church. | Ireland | Qualitative | Data collection:  Interviews;  Data analysis:  IPA. | Conflict /cognitive dissonance /tension |
| **Total of studies:** | N.A. | | N.A. | | Catholic: 78 studies;  Unspecified Christian affiliations: 58  LDS: 29;  Christian Protestant: 26;  Baptist: 18;  Evangelical: 18;  Methodist: 16;  Presbyterian: 15;  Nondenominational Christian: 12;  Lutheran: 9;  Pentecostal: 9;  United Church of Christ: 9;  SDA: 8;  Episcopal: 7;  Jehovah´s Witness: 5;  Anglican: 4;  Black Church: 4;  Metropolitan Community Church: 4. | N.A. | USA: 121  studies;  UK: 3;  Australia: 2;  Canada: 2; Italy: 2;  Portugal: 2;  Poland: 2;  Ireland: 2;  Hong Kong: 1;  Mexico: 1;  Italy and Spain: 1;  Brazil: 1; Norway: 1; Austria: 1; Philippines: 1. | Qualitative: 84 studies;  Quantitative: 53 studies;  Mixed methods: 6 studies;  Theoretical: 7 studies. | Data collection:  Individual interviews (*n* = 66 out of 84 qualitative studies);  Online surveys (*n* = 59 out of 59 quantitative and mixed method studies).  Data analysis:  Content or thematic analyses (n = 36 out of 84 qualitative studies);  Grounded Theory (n = 14 out of 84 qualitative studies);  Interpretative Phenomenological Analysis (IPA): (n = 10 out of 84 qualitative studies). | Identity conflict / incongruity / Tension / ambivalence / cognitive dissonance: 81 studies;  Minority Stress Theory: 29;  Intersectionality: 25;  Identity integration: 23;  Religious/Spiritual Struggles: 11;  Resilience: 7;  Feminist Theory: 5;  Queer Theory: 5;  Positive Psychology frameworks: 3; Stress and Coping Model (based on Park and Folkman, 1997): 3;  Empowerment theory: 2;  Religious Trauma: 2;  Ambiguous Loss  Theory: 1;  Causal Pathways Theory: 1. |

1. Some studies just report sexual orientation, and others just report gender identity. We collected the data for each study accordingly. [↑](#footnote-ref-1)
2. Although some studies include religions other than Christianity or participants with no religion, we only extract data on Christian affiliations, as Christianity is our interest. Some studies do not report the number of participants in each Christian confession. We collected the data for each study accordingly.

   ^3^ According to Petters et al. (2021). [↑](#footnote-ref-2)
