# Supplementary material for: Being Lesbian, Gay, Bisexual, Trans, Queer, or Intersex (LGBTQI) and Christian: A Scoping Review of Theories and Constructs in Psychological Research
Source: Int J Sex Health. 2024 Apr 11;36(4):439–63. doi: 10.1080/19317611.2024.2331806 (PMC11562956; doi:10.1080/19317611.2024.2331806)
Supplement: Supplemental Material [file WIJS_A_2331806_SM8387.docx]

**Title: Being Lesbian, Gay, Bisexual, Trans, Queer, or Intersex (LGBTQI) and Christian:**

**A Scoping Review of Theories and Constructs in Psychological Research**

**Supplementary Material 1: Full Search Strategy**

**1. Initial First General Search to Identify Relevant Articles on the Topic**

Table 1. Initial general search strategy in EBSCOhost

| **#** | **Search Term** | **Results** |
| --- | --- | --- |
| 1 | (#lgbtq or #lesbian or #gay or #homosexual or #bisexual or #transgender or #queer or #sexual minority ) AND #christianity AND #psychology) | 271 |
| 2 | #1 (Limit to 2012-2022) | **232** |
| 3 | (#lgbtq or #lesbian or #gay or #homosexual or #bisexual or #transgender or #queer or #sexual minority ) AND #christianity AND #psychology research) | 8 (Duplicates) |
| 4 | (#lgbtq or #lesbian or #gay or #homosexual or #bisexual or #transgender or #queer or #sexual minority ) AND #christianity AND #psychological well-being) | 6  (Duplicates) |

**2. Refine Search Strategy**

Table 2. Collecting and dividing the keywords of the relevant articles found in the initial general search according to the three search elements

| **1^st^ Search Element Keywords**  (LGBTQI+ people related keywords) | **2^nd^ Search Element**  (Religion and Christianity related keywords) | **3^rd^ Search Element**  (Psychology related keywords) | **Others** |
| --- | --- | --- | --- |
| Lesbians  Lesbian (6) | Non-affirming religious settings | Homophobia (2)  Homonegativity | Positive outcomes |
| Gay men  Gay (6)  Latino gay  Male homosexuality | Affirming religious settings  Affirming congregations | Internalized homophobia  Internalized homonegativity | Negative outcomes |
|  | Religious homophobia | Stigma | Anti-gay  sentiment |
| Bisexuals  Bisexual | Religiosity (5) | Spiritual resilience | Psychosexual behavior |
| Homosexuality (4) | Religiousness | Reconciliation | Cultural competence |
| Sexual minority (3)  Sexual- and gender-minority | Religion (19) | Spiritual struggles | Spiritually sensitive therapy |
| Christian sexual minority | Christianity (5) | Self-esteem (2) | Power/knowledge |
| Sexual orientation (3) | Christian upbringing | Spatial coping strategy | Thematic analysis |
| MTF transgender | Church | Minority stress theory | Adolescents  Adolescent  Adolescence |
| Transgender (7) | Christian colleges | Conflict | Health  Mental illness  Mental disorders |
| Gender identity (3) | Religious abuse | Identity conflict (5)  Identity conflicts (2) | Images of God |
| Christian (4) | Faith (3) | Religious identity/sexual orientation identity conflict | Social pressure for religious  conformity |
| Religion transgender Christians | Religious beliefs | Identity integration (2) |  |
| LGB identity | Religious disaffiliation | Ambiguous loss theory | Group norms |
| Sexual identity (3) | Spiritual | Intersectionality | Coming out |
| Black MSM | Muslim | (narrative therapy) Re-authoring  (technique) | Family |
| Mormon | Spirituality (8) | Empowerment | Life course |
| Catholic |  | Cognitive dissonance | Loss  Grief |
| LGB (2)  LGBT (4)  LGBT+  LGBTQ (2)  LGBTQIA+ |  | Conflict resolution strategies | Childhood |
| Religious Identity (3) |  | Psychological  well-being | Children |
| Gender diverse |  | Traffic light model |  |
| Queer (2) |  | Mental health (7) | Social identity |
| Affectional orientation |  | Affirmative therapy | Personal identity |
| Gender nonconforming |  | Feminist  phenomenology | Youth |
| Homosexual |  |  | Systematic review |
| Sexual orientation identity |  |  | Negative  attitudes towards  homosexuality |
| Intersex |  |  | Sexual identity development |
| Sexually diverse |  |  | Religious  identity development |
|  |  |  | Identity development |
|  |  |  | Identity (2) |
|  |  |  | Shame |
|  |  |  | Malta |
|  |  |  | Palermo |
|  |  |  | Ireland |
|  |  |  | Culture  Cultural context |
|  |  |  | Asian sexuality |
|  |  |  | Sexuality (7) |

**3. Matching the most used keywords with indexed terms in APA Thesaurus, Academic Search Ultimate, and Psychological and Behavioral Sciences for 1^st^ Search Element, 2^nd^ Search Element, and 3^rd^ Search Element**

Table 3. Keywords and corresponding index terms in APA Thesaurus, Academic Search Ultimate, and Psychological and Behavioral Sciences for the 1^st^ Search Element

| **1^st^ Search Element Keywords**  (LGBTQI+ people) | **APA Thesaurus of Psychological Index Terms** | **Academic Search Ultimate Subject Terms** | **Psychological and Behavioral Sciences Collection - Subjects** |
| --- | --- | --- | --- |
| **LGBT** | **-** LGBTQ  Broader terms:  Gender identity  Sexual orientation  Asexuality  Bisexuality  Homosexuality  Intersex  Transgender | - LGBTQ + people | **-** LGBTQ + people^[[1]](#footnote-1)^  Broader terms:  Sexual minorities  Narrower terms:  Bisexuals  Christian LGBTQ+ people  Gay people  Lesbians  Minority LGBTQ people  Transgender people  Related terms:  Church work with LGBTQ+ people  LGBTQ+ identity  Sexual orientation |
| **Transgender** | - Transgender  Broader terms:  - Transsexualism (1973 to 2009)  - LGBTQ  - Gender expression  - Sexual minority groups | The term alone does not exist, but exist:  - Catholic transgender people  Broader terms:  - Catholic LGBTQ+ people  - Christian transgender people  - Catholic bisexuals  - Catholic gay people | The term alone does not exist, but exist:  - Christian transgender people  Broader terms:  - Christian LGBTQ+ people  - Transgender people  Narrower terms:  - Catholic transgender people  - Protestant transgender people  Related terms:  - Christian bisexuals  - Christian gay people |
| **Gay** | - Male homosexuality | The term alone does not exist, but exist:  - LGBTQ+ studies | The term alone does not exist, but exist:  - LGBTQ+ studies (instead of gay & lesbian studies)  - Christian gay men  - Christian gay people  Broader terms:  - Christian LGBTQ+ people  - Christians  - Gay people  Narrower terms:  - Catholic gay people  - Christian gay men  - Christian lesbians  - Mormon gay people  - Protestant gay people  Related terms:  Christian bisexuals  Christian transgender people |
| **Lesbian** | **-** Lesbianism | The term alone does not exist, but exist:  - LGBTQ+ studies | The term alone does not exist, but exist:  Lesbian clergy  Related terms:  - Religious life of lesbians  **- Christian lesbians**  Broader terms:  - Christian gay people  - Christian women  - Lesbians  Narrower terms:  - Catholic lesbians  - Mormon lesbians  - Protestant lesbians |
| **Gender identity** | - Gender identity  - Gender nonbinary  - Gender nonconforming  - LGBTQ | - Gender identity  Narrower terms:  - Gender nonconformity  - Intersexual identity  - Sexual diversity  - Third gender  - Transgender identity  - Transgenderism  - Transsexualism  Related terms:  - Gender identity in the Bible  - Gender nonconforming people  - LGBTQ+ identity  - Questioning people  - Sexual orientation  - Sexual orientation identity  - Tomboys  - Queer theory | The same terms as in Academic Search Ultimate Subject Terms *(on the left)* |
| **Queer** | - LGBTQ | - Queer^[[2]](#footnote-2)^  - LGBTQ+ people | The term alone does not exist, but exist:  - Sexual minority community  Narrower terms:  - LGBTQ+ communities  - Sexual minorities  - Sexual minority men  - Sexual minority women  - Sexual minority youth  - Sexual orientation  - Sexual orientation & religion  - Sexual orientation identity:  Narrower terms:  - Identity of sexual minorities  - LGBTQ+ identity  - Sexual diversity  Related terms:  - Gender identity  - Sexual orientation |
| **Sexual orientation** | - Sexual orientation  - LGBTQ | - Sexual orientation^[[3]](#footnote-3)^  Narrower terms:  - Bisexuality  - Homosexuality  - Lesbianism  - Pansexuality  - Sexual fluidity  Related terms:  - Gender identity  - LGBTQ+ people  - Questioning people  - Sexual orientation & religion  - Sexual orientation identity^[[4]](#footnote-4)^ (NT: identity of sexual minorities; LGBTQ+ identity; sexual diversity. RT: Gender identity; Sexual orientation) | The same terms as in Academic Search Ultimate Subject Terms *(on the left)* |
| **Sexual minority** | - Sexual minority groups  - Sexual orientation | The term alone does not exist, but exist:  - Sexual minority men  Broader terms:  - Sexual minorities  - Bisexual men  - Gay men  - Trans    Narrower terms:  - Men  Related terms:  - Men who have sex with men  - Sexual minority women  Broader terms:  - Sexual minorities  Narrower terms:  - Bisexual women  - Lesbians  - Trans women  Related terms:  - Women who have sex with women | The same terms as in Academic Search Ultimate Subject Terms *(on the left)* |
| **Homosexuality** | - Homosexuality  Broader terms:  - LGBTQ  Narrower terms:  - Lesbianism  - Male homosexuality  Related terms:  - Sexual minority groups | - Homosexuality^[[5]](#footnote-5)^  Narrower terms:  - Bible & homosexuality^[[6]](#footnote-6)^  - Homosexuality in the Bible  - Lesbianism  - Male homosexuality  Related terms:  - Bisexuality  - Gay men  - Gay people  - Transgenderism | The same terms as in Academic Search Ultimate Subject Terms *(on the left)* |
| **Bisexual** | The term alone does not exist, but exist:  - Catholic bisexuals  Broader Terms:  - Catholic LGBTQ+ people  - Christian bisexuals  Related Terms:  - Catholic gay people;  - Catholic transgender people  When search bisexuality:  - Bisexuality  Broader terms:  - LGBTQ  Related terms:  - Lesbianism  - Male homosexuality  - Sexual minority groups | The term alone does not exist, but exist:  - Catholic bisexuals  Broader terms:  - Catholic LGBTQ+ people  - Christian bisexuals  Related terms:  - Catholic gay people  - Catholic transgender people | The term alone does not exist, but exist:  - Bisexual identity  Broader Terms:  - LGBTQ+ identity  Narrower Terms:  - Bisexual men´s identity;  - Bisexual women´s identity  Related Terms: Gay identity  - Bisexual men  Broader Terms:  - Bisexuals  - Sexual minority men  Related Terms:  - Bisexual men´s identity  - Bisexual teenagers  - Bisexual women  - Men who have sex with men  - Bisexual men´s identity  - Bisexual teenagers  - Bisexual women  - Bisexual women´s identity  - Bisexuality  - Bisexuality & religion |

Table 4. Keywords and corresponding index terms in in APA Thesaurus, Academic Search Ultimate, and Psychological and Behavioral Sciences for the 2^nd^ Search Element

| **2^nd^ Search Element Keywords**  (Religion and Christianity) | **APA Thesaurus of Psychological Index Terms**  (includes APA databases) | **Academic Search Ultimate Subject Terms** | **Psychological and Behavioral Sciences Collection - Subjects** |
| --- | --- | --- | --- |
| **Religiosity** | - Religiosity  - Religious beliefs  - Religious affiliation  - Religious experiences  - Religious fundamentalism  - Religious practices  - Religious prejudices | - Religiousness | - Religiousness |
| **Religion** | - Religion  (Scope note: Conceptually broad term. Use a more specific term if possible.)  - Religious beliefs  - Religious experiences  - Religious practices  - Religious prejudices | - Religion^[[7]](#footnote-7)^ | The same terms as in Academic Search Ultimate Subject Terms *(on the left)* |
| **Christianity** | - Christianity  Broader terms:  - Religious affiliation  Narrower terms:  - Protestantism  - Roman Catholicism  Related terms:  - Bible  - Christians | - Christianity^[[8]](#footnote-8)^  Narrower terms:  - Church  Related terms:  - Christian LGBTQ+ people | The same terms as in Academic Search Ultimate Subject Terms *(on the left)* |
| **Affirming religious settings** | The term does not exist, nor other similar. | The term does not exist, nor “religious settings” as a broader term. We found:  - Religious communities  Narrower terms:  - Christian communities  Related terms:  **-** Religious groups | The same terms as in Academic Search Ultimate Subject Terms *(on the left)* |
| **Nonaffirming religious settings** | The term does not exist, nor other similar. | The term does not exist, nor other similar. | The term does not exist, nor other similar. |
| **Christian upbringing** | The term does not exist. The search turns to words like “Christianity” and “Christians”, as we already included in our search strategy. | The term does not exist. We found:  **-** Christian life (Scope note: Here are entered works descriptive of, or seeking to foster, personal Christian religious and devotional life. Use only if a narrower term does not apply (e.g. "Christian ethics" or "Piety"). [EPC] | The same terms as in Academic Search Ultimate Subject Terms *(on the left)* |
| **Faith** | - Faith  Broader terms:  - Religious beliefs | - Faith  Narrower terms:  - Faith (Christianity) | The same terms as in Academic Search Ultimate Subject Terms *(on the left)* |

Table 5. Keywords and corresponding index terms in in APA Thesaurus, Academic Search Ultimate, and Psychological and Behavioral Sciences for the 3^rd^ Search Element

| **3^rd^ Search Element Keywords**  (Psychology) | **APA Thesaurus of Psychological Index Terms**  (includes APA databases) | **Academic Search Ultimate Subject Terms** | **Psychological and Behavioral Sciences Collection - Subjects** |
| --- | --- | --- | --- |
| **Identity conflict** | The term does not exist, but there is one similar:  - Identity crisis (Scope note: A period of personal adjustment marked by role experimentation, changing values, and emotional adjustment.)  Related terms:  - Self-concept (Narrower terms: self-esteem; Related terms: Gender identity)  - Stress  (Narrower terms: Minority stress, Psychological stress) | The term does not exist | The term does not exist |
| **Identity Integration** | The term does not exist | The term does not exist | The term does not exist |
| **Conflict resolution** | - Conflict resolution  Scope note: Process of reducing or removing antagonisms among individuals, groups, organizations, or political entities. | The term does not exist, but there are two similar:  - Conflict management  (Here are entered works which discuss the process of reducing, resolving or suppressing conflict in social and organizational settings producing constructive rather than destructive results.) [EPC]  - Conflict management in the Bible | The term does not exist, but there are two similar:  - Conflict management  (Here are entered works which discuss the process of reducing, resolving or suppressing conflict in social and organizational settings producing constructive rather than destructive results.) [EPC]  - Reconciliation  (Here are entered general works on the restoration of an amicable relationship between disagreeing or conflicting persons or groups. Works on the process by which two or more parties with differing goals reach an agreement about a cooperative resolution are entered under "Negotiation.") [EPC]  Related term:  - Religious dissenters (Here are entered works on persons who disagree with the authority of an established religious body, especially Protestants who reject the Church of England.) [EPC] |
| **Psychological well-being** | The term does not exist. | - Psychological well-being  Broader term: Well-being  Related term: Psychological adaptation | The same terms as in Academic Search Ultimate Subject Terms *(on the left)* |
| **Self-esteem** | - Self-esteem | - Self-esteem  (Here are entered works on the subjective feeling of self-worth built from the respect and sense of worth reflected back on the person from significant others. Works on a person's acceptance of their own positive and negative traits, resulting in a happy, satisfied person, are entered under "Self-acceptance." Works on a person's confidence or faith in their abilities are entered under "Self-confidence.”)[EPC] | The same terms as in Academic Search Ultimate Subject Terms *(on the left)* |
| **Homophobia** | The term does not exist. The suggestion is to use Homosexuality (Attitudes Toward) | - Homophobia  - Homophobia -- Religious aspects  (Here are entered works on religious views concerning aversion to or discrimination against gay men or lesbians.) [EPC] | The same terms as in Academic Search Ultimate Subject Terms *(on the left)* |
| **Psychology**^[[9]](#footnote-9)^ | **-** Psychology  - Psychological theories | **-** Psychology  - Psychology & religion (Scope note: Here are entered works about mutual relationships between psychology and religion in general; use only if a more specific term does not apply. Works on the psychology of religion are entered under "Religious psychology." [EPC]  Narrower terms:  - Psychiatry & religion  - Religious psychology (Narrower terms: religious experience; religious identity; Related terms: religious influence) | The same terms as in Academic Search Ultimate Subject Terms *(on the left)* |
| **Identity conflict theory/ies**^[[10]](#footnote-10)^ | The term does not exist. The search turns to words like “identity crisis” as we already included in our search strategy. | The term does not exist. The search turns to words like “identity crises (Psychology)”. | The term does not exist. The search turns to words like “identity crises (Psychology)”, and Identity of sexual minorities (Broader terms: sexual orientation identity; Narrower terms: LGBTQ+ identity) |
| **Conflict theories** | The term does not exist, nor do other similar words. | The term does not exist. The search goes to “conflict theory”, which is not about what we refer to in this context. | The same terms as in Academic Search Ultimate Subject Terms *(on the left)* |
| **Minority Stress theory** | The complete term does not exist. We found “Minority stress”, as we already included in our search strategy. | The same terms as in APA Thesaurus of Psychological Index Terms *(on the left)* | The same terms as in APA Thesaurus of Psychological Index Terms *(on the left)* |
| **Empowerment** (theory) | - Empowerment (Scope note: Promotion or attainment of autonomy and freedom of choice for individuals or groups.) | The term does not exist, nor do other similar words. | The same terms as in Academic Search Ultimate Subject Terms *(on the left)* |
| **Traffic light model** | The term does not exist, nor do other similar words. | The term does not exist, nor do other similar words. | The term does not exist, nor do other similar words. |
| **Affirmative Therapy** | - Affirmative Therapy | The term does not exist, nor do other similar words. | The term does not exist, nor do other similar words. |
| **Mental Health** | - Mental Health | - Mental Health  Scope note: Here are entered general works on psychological and emotional states, and on the branch of medicine concerned with the attainment and continuation of good psychological health. Use only if a narrower term does not apply (e.g., "Child mental health," or "Stress management"). [EPC]  - Mental Health & religion  Related terms:  - Mental illness & religion  Related terms:  Religious psychology | The same terms as in Academic Search Ultimate Subject Terms *(on the left)* |

**4. Selecting the keywords for refining the search strategy**^[[11]](#footnote-11)^

Table 6. The selected keywords to incorporate in the search strategy

|  | **1^st^ Search Element**  (LGBQI+ people) | **2^nd^ Search Element**  (Religion and Christianity) | **3^rd^ Search Element**  (Psychology) |
| --- | --- | --- | --- |
| **Broader search terms** | - Sexual minority/ies  - LGBTQ+ people  - GLBTQ  - Homosexuality/ies  - Lesbianism  - Bisexuality  - Transgenderism  - Transsexualism  - Transsexuality  - Gender nonconformity  - Gender nonbinary  - Gender diverse  - Sexually diverse  - Gender dysphoria  - Gender queer  - Same gender loving  - Same sex attracted | - Religiosity  - Religiousness  - Christianity  - Christians  - Church  - Religious affiliation  - Religious practices  - Religious prejudices  - Religious beliefs  - Religious experiences  - Religious communities  - Religious groups  - Religious influence | - Psychology  - Religious Psychology  - Psychology theories  - Empowerment (theory)  - Identity crisis  - Self-concept  - Self-esteem  - Stress  - Psychological well-being  - Psychological adaptation |
| **Narrower search terms** | - Bisexuals  - Homosexual/s  - Gay / Gays  - Lesbigay  - Male homosexuality  - Men who have sex with men (MSM)  - Lesbians  - Women who have sex with women (WSW)  - Women loving women  - Transgender/s  - Trans people  - Trans person  - Trans/sexual  - Gender nonconforming  - Intersex  - Queer  - F2M  - Female-to-male  - M2F  - Male-to-female  - Trans female  - Trans male  - Trans men  - Trans woman | - Christian communities  - Christian LGBTQ+ people  - Christian transgender people  - Christian gay people  - Christian lesbians  - Christian bisexuals  - Mormon gay people  - Mormon lesbians  - Protestant transgender people  - Protestant gay people  - Protestant lesbians | - Identity conflict (theory)  - Conflict (theories)  - Identity integration  - Conflict resolution  - Conflict management (in the Bible)  - Minority Stress (theory)  - Traffic light model  - Affirmative Therapy |
| **Related search terms** | - Sexual orientation  - Gender identity  - Gender reassign  - Gender change  - Sex change  - Sex transition  - Sexuality | - Religious fundamentalism  - Reconciliation  - Loss  - Grief | - Mental Health |

**5. Refining search strategy**

Table 7. Refined search strategy in EBSCOhost (Academic Search Ultimate, Fonte Acadêmica, APA PsycInfo, APA PsycArticles, and Psychology and Behavioral Sciences Collection)

| **#** | **Search Term** | **Results** |
| --- | --- | --- |
| **1** | (#sexual minority or #lgbtq or #glbtq or #homosexuality or #homosexual or #gay or #male homossexuality or #men who have sex with men or #lesbianism or #lesbians or #women who have sex with women or #women loving women or #bisexuality or #bisexuals or # transgender or #trans or #transgenderism or #transsexualism or #transsexuals or #tanssexuality or #gender nonconforming or #F2M or #female-to-male or #M2F or #male-to-female or #trans male or #trans female or #trans men or #trans women or #intersex or #queer) AND (religiosity or #religiousness or #christianity or #christians or #church or #religion affiliation or #religious practices or #religious prejudices or #religious beliefs or #religious experiences) AND (#psychology or #religious psychology or #psychology theories or #identity conflict or #minority stress)  (Limit to 2012-2022)  (Limit to Academic Journals and Dissertations) | **1226** |
| 2 | (#Christian LGBTQ+ people or #Christian transgender people or #Christian gay people or #Christian lesbians or #Christian bisexuals or #Mormon gay people or #Mormon lesbians or #Protestant transgender people or #Protestant gay people or #Protestant lesbians) AND (#psychology or #religious psychology or #psychology theories or #Identity conflict or #Identity integration or #Conflict resolution or #Conflict management in the Bible or #Minority Stress or #Traffic light model or #Affirmative therapy)  (Limit to 2012-2022) | 38  (all duplicates from the 1^st^ search) |

Table 8. Search Strategy in Web of Science

| **#** | **Search Term** | **Results** |
| --- | --- | --- |
| 1 | (((ALL=(sexual minority OR lgbtq or glbtq OR homosexuality OR homosexual OR gay OR male homossexuality OR men who have sex with men OR lesbianism OR lesbians OR women who have sex with women OR women loving women OR bisexuality OR bisexuals OR transgender OR trans OR transgenderism OR transsexualism OR transsexuals OR tanssexuality OR gender nonconforming OR F2M OR female-to-male OR M2F OR male-to-female OR trans male OR trans female OR trans men OR trans women OR intersex or queer)) AND ALL=(religiosity OR religiousness OR christianity OR christians OR church OR religion affiliation OR religious practices OR religious prejudices OR religious beliefs OR religious experiences)) AND ALL=(psychology OR religious psychology OR psychology theories OR identity conflict OR minority stress)) AND PY=(2012-2022) | 505 |
| 2 | ((((ALL=(sexual minority OR lgbtq or glbtq OR homosexuality OR homosexual OR gay OR male homossexuality OR men who have sex with men OR lesbianism OR lesbians OR women who have sex with women OR women loving women OR bisexuality OR bisexuals OR transgender OR trans OR transgenderism OR transsexualism OR transsexuals OR tanssexuality OR gender nonconforming OR F2M OR female-to-male OR M2F OR male-to-female OR trans male OR trans female OR trans men OR trans women OR intersex or queer)) AND ALL=(religiosity OR religiousness OR christianity OR christians OR church OR religion affiliation OR religious practices OR religious prejudices OR religious beliefs OR religious experiences))) AND PY=(2012-2022) AND SU=(psychology)) | 708 |
| **3** | ((((ALL=(sexual minority OR lgbtq or glbtq OR homosexuality OR homosexual OR gay OR male homossexuality OR men who have sex with men OR lesbianism OR lesbians OR women who have sex with women OR women loving women OR bisexuality OR bisexuals OR transgender OR trans OR transgenderism OR transsexualism OR transsexuals OR tanssexuality OR gender nonconforming OR F2M OR female-to-male OR M2F OR male-to-female OR trans male OR trans female OR trans men OR trans women OR intersex or queer)) AND ALL=(religiosity OR religiousness OR christianity OR christians OR church OR religion affiliation OR religious practices OR religious prejudices OR religious beliefs OR religious experiences))) AND PY=(2012-2022) AND SU=(psychology)) and Psychology Multidisciplinary or Psychology Clinical or Psychology Social or Psychology Applied or Psychology Developmental or Psychology or Psychology Educational or Psychology Psychoanalysis or Psychology Experimental or Psychology Biological or Psychology Mathematical (Web of Science Categories) | **708** |

Table 10. Search Strategy in Scopus

| **#** | **Search Term** | **Results** |
| --- | --- | --- |
| 1 | ( sexual AND minority OR lgbtq OR glbtq OR homosexuality OR homosexual OR gay OR male AND homossexuality OR men AND who AND have AND sex AND with AND men OR lesbianism OR lesbians OR women AND who AND have AND sex AND with AND women OR women AND loving AND women OR bisexuality OR bisexuals OR transgender OR trans OR transgenderism OR transsexualism OR transsexuals OR tanssexuality OR gender AND nonconforming OR f2m OR female-to-male OR m2f OR male-to-female OR trans AND male OR trans AND female OR trans AND men OR trans AND women OR intersex OR queer ) AND ( religiosity OR religiousness OR christianity OR christians OR church OR religion AND affiliation OR religious AND practices OR religious AND prejudices OR religious AND beliefs OR religious AND experiences ) AND ( psychology OR religious AND psychology OR psychology AND theories OR identity AND conflict OR minority AND stress ) | 104 |
| 2 | ( sexual AND minority OR lgbtq OR glbtq OR homosexuality OR homosexual OR gay OR male AND homossexuality OR men AND who AND have AND sex AND with AND men OR lesbianism OR lesbians OR women AND who AND have AND sex AND with AND women OR women AND loving AND women OR bisexuality OR bisexuals OR transgender OR trans OR transgenderism OR transsexualism OR transsexuals OR tanssexuality OR gender AND nonconforming OR f2m OR female-to-male OR m2f OR male-to-female OR trans AND male OR trans AND female OR trans AND men OR trans AND women OR intersex OR queer ) AND ( religiosity OR religiousness OR christianity OR christians OR church OR religion AND affiliation OR religious AND practices OR religious AND prejudices OR religious AND beliefs OR religious AND experiences ) AND ( psychology OR religious AND psychology OR psychology AND theories OR identity AND conflict OR minority AND stress ) AND PUBYEAR > 2011 AND PUBYEAR < 2023 AND ( LIMIT-TO ( SUBJAREA , "PSYC" ) OR LIMIT-TO ( SUBJAREA , "SOCI" ) OR LIMIT-TO ( SUBJAREA , "MEDI" ) OR LIMIT-TO ( SUBJAREA , "HEAL" ) ) AND ( LIMIT-TO ( DOCTYPE , "ar" ) OR LIMIT-TO ( DOCTYPE , "re" ) ) | 13 |
| 3 | lgbt AND christianity AND psychology AND PUBYEAR > 2011 AND PUBYEAR < 2023 AND PUBYEAR > 2011 AND PUBYEAR < 2023 AND ( LIMIT-TO ( SUBJAREA , "PSYC" ) ) AND ( EXCLUDE ( DOCTYPE , "bk" ) OR EXCLUDE ( DOCTYPE , "ch" ) OR EXCLUDE ( DOCTYPE , "re" ) OR EXCLUDE ( DOCTYPE , "no" ) OR EXCLUDE ( DOCTYPE , "ed" ) ) | 186 |
| **4** | ( TITLE-ABS-KEY ( sexual AND minority ) OR TITLE-ABS-KEY ( lgbtq ) OR TITLE-ABS-KEY ( glbtq ) OR TITLE-ABS-KEY ( homosexuality ) OR TITLE-ABS-KEY ( homosexual ) OR TITLE-ABS-KEY ( gay ) OR TITLE-ABS-KEY ( male AND homossexuality ) OR TITLE-ABS-KEY ( men AND who AND have AND sex AND with AND men ) OR TITLE-ABS-KEY ( lesbianism ) OR TITLE-ABS-KEY ( lesbians ) OR TITLE-ABS-KEY ( women AND who AND have AND sex AND with AND women ) OR TITLE-ABS-KEY ( women AND loving AND women ) OR TITLE-ABS-KEY ( bisexuality ) OR TITLE-ABS-KEY ( bisexuals ) OR TITLE-ABS-KEY ( transgender ) OR TITLE-ABS-KEY ( trans ) OR TITLE-ABS-KEY ( transgenderism ) OR TITLE-ABS-KEY ( transsexualism ) OR TITLE-ABS-KEY ( transsexuals ) OR TITLE-ABS-KEY ( transsexuality ) OR TITLE-ABS-KEY ( gender AND nonconforming ) OR TITLE-ABS-KEY ( f2m ) OR TITLE-ABS-KEY ( female AND to AND male ) OR TITLE-ABS-KEY ( m2f ) OR TITLE-ABS-KEY ( male AND to AND female ) OR TITLE-ABS-KEY ( trans AND male ) OR TITLE-ABS-KEY ( trans AND female ) OR TITLE-ABS-KEY ( trans AND men ) OR TITLE-ABS-KEY ( trans AND women ) OR TITLE-ABS-KEY ( intersex ) OR TITLE-ABS-KEY ( queer ) AND TITLE-ABS-KEY ( religiosity ) OR TITLE-ABS-KEY ( religiousness ) OR TITLE-ABS-KEY ( christianity ) OR TITLE-ABS-KEY ( christians ) OR TITLE-ABS-KEY ( church ) OR TITLE-ABS-KEY ( religion AND affiliation ) OR TITLE-ABS-KEY ( religious AND practices ) OR TITLE-ABS-KEY ( religious AND prejudices ) OR TITLE-ABS-KEY ( religious AND beliefs ) OR TITLE-ABS-KEY ( religious AND experiences ) AND TITLE-ABS-KEY ( psychology ) OR TITLE-ABS-KEY ( religious AND psychology ) OR TITLE-ABS-KEY ( psychology AND theories ) OR TITLE-ABS-KEY ( identity AND conflict ) OR TITLE-ABS-KEY ( minority AND stress ) ) AND PUBYEAR > 2011 AND PUBYEAR < 2023 AND ( LIMIT-TO ( SUBJAREA , "PSYC" ) ) AND ( LIMIT-TO ( DOCTYPE , "ar" ) ) | **1019** |

1. Here are entered general studies regarding people who identify themselves as lesbian, gay, bisexual, transgender, and queer or questioning. [↑](#footnote-ref-1)
2. Studies with participants who identify themselves as queer are entered under "LGBTQ+ people." Studies on philosophical or critical thought regarding gay, lesbian, bisexual, transgender, or queer or questioning viewpoints are entered under "Queer theory." [↑](#footnote-ref-2)
3. Here are entered general works on the inclination or interest of a person's sexual desires. Use only if a narrower term does not apply (e.g. "Heterosexuality" or "Lesbianism") [EPC] [↑](#footnote-ref-3)
4. Here are entered works on a person's inner concept of their own sexual orientation. Works on the inclination or interest of a person's sexual desires are entered under "Sexual orientation." Works on a person's inner concept of self as belonging to one particular sex are entered under "Gender identity." Use only if a narrower term does not apply (e.g., "Gay people -- Identity"). [EPC] [↑](#footnote-ref-4)
5. Here are entered works on sexual attraction exclusively toward others of the same sex. Use only if a narrower term does not apply (e.g., "Lesbianism," or "Male homosexuality"). Works on persons attracted exclusively to members of the same sex are entered under "Gay people" or an appropriate narrower term. [EPC] [↑](#footnote-ref-5)
6. Here are entered works the mutual relationship between the Bible and homosexuality. [EPC] [↑](#footnote-ref-6)
7. Here are entered general works on the various ways individuals explore fundamental religious questions outside of organized religion, including beliefs about a deity, how to live a good life, and what is the meaning of existence. Works on the major world religions are entered under "Religions." Use only if a narrower term does not apply (e.g., "Animism" or "Holy, The"). [EPC] [↑](#footnote-ref-7)
8. Here are entered works on the Christian religion including its origin, beliefs, practices and influence, treated collectively. Works on the institutional history of the church are entered under "Church history." Use only if a narrower term does not apply (e.g. "Christian sects" or "Ecclesiastical law"). [EPC] [↑](#footnote-ref-8)
9. We did not find this term in the keywords of the articles found in the first general search, but we added it here because Psychology is our context of research. [↑](#footnote-ref-9)
10. We did not find this term in the keywords of the articles in the first general search, but we added it here because we found this theory/approach in several studies. [↑](#footnote-ref-10)
11. We also acknowledge and complete with what Lee et al. (2016) found regarding LGBT search terminology. [↑](#footnote-ref-11)
